# The Circadian Clock Coordinates Ribosome Biogenesis

**DOI:** 10.1371/journal.pbio.1001455

**Published:** 2013-01-03

**Authors:** Céline Jouffe, Gaspard Cretenet, Laura Symul, Eva Martin, Florian Atger, Felix Naef, Frédéric Gachon

**Affiliations:** 1 Department of Pharmacology and Toxicology, University of Lausanne, Lausanne, Switzerland; 2 The Institute of Bioengineering, School of Life Sciences, Ecole Polytechnique Fédérale de Lausanne, Lausanne, Switzerland; Texas A&M, United States of America

## Abstract

Biological rhythms play a fundamental role in the physiology and behavior of most living organisms. Rhythmic circadian expression of clock-controlled genes is orchestrated by a molecular clock that relies on interconnected negative feedback loops of transcription regulators. Here we show that the circadian clock exerts its function also through the regulation of mRNA translation. Namely, the circadian clock influences the temporal translation of a subset of mRNAs involved in ribosome biogenesis by controlling the transcription of translation initiation factors as well as the clock-dependent rhythmic activation of signaling pathways involved in their regulation. Moreover, the circadian oscillator directly regulates the transcription of ribosomal protein mRNAs and ribosomal RNAs. Thus the circadian clock exerts a major role in coordinating transcription and translation steps underlying ribosome biogenesis.

## Abstract 2

The authors identify a new role of the circadian clock in coordinating mRNA translation during ribosome biogenesis, a key process for cell metabolism.

## Author Summary

Most living organisms on earth present biological rhythms that play a fundamental role in the coordination of their physiology and behavior. The discovery of the molecular circadian clock gives important insight into the mechanisms involved in the generation of these rhythms. Indeed, this molecular clock orchestrates the rhythmic transcription of clock-controlled genes involved in different aspects of metabolism, for example lipid, carbohydrate, and xenobiotic metabolisms in the liver. However, we show here that the circadian clock could also exert its function through the coordination of mRNA translation. Namely, the circadian clock influences the temporal translation of a subset of mRNAs by controlling the expression and activation of translation initiation factors, as well as the clock-dependent rhythmic activation of signaling pathways involved in their regulation. These rhythmically translated mRNAs are mainly involved in ribosome biogenesis, an energy consuming process, which has to be gated to a period when the cell resources are less limited. Moreover, the role of the circadian oscillator in this process is highlighted by its direct regulation of the transcription of ribosomal protein mRNAs and ribosomal RNAs. Thus our findings suggest that the circadian clock exerts a major role in coordinating transcription and translation steps underlying ribosome biogenesis.

## Introduction

Circadian rhythms in behavior and physiology reflect the adaptation of organisms exposed to daily light-dark cycles. As a consequence, most aspects of metabolism and behaviour are under the control of these rhythms [1]. At a molecular level, in all the studied species, the rhythmic expression of the genes involved originates in the network of interconnected transcriptional and translational feedback loops [2]. In mammals, the heterodimer composed of BMAL1 and its partners CLOCK or NPAS2 is a transcriptional activator that regulates transcription of the *Period* (*Per*) and *Cryptochrome* (*Cry*) genes that code for repressors of BMAL1 heterodimer activity, thus closing a negative feedback loop that generates rhythms of approximately 24 h [1],[2]. Many efforts during the last decade have characterized rhythmically expressed genes and delimit the impact of the circadian clock on physiology. Numerous circadian transcriptome studies in different species and organs show that approximately 10% of the genes are rhythmically expressed. The functions of these genes established the role of the circadian clock in temporally gating rhythmic physiology [1],[3]. However, increasing evidence suggests that transcriptional mechanisms are not sufficient to explain numerous observations. For example, it has been shown that many oscillating proteins in mouse liver are encoded by constantly expressed mRNAs [4].

Interestingly, among the rhythmically expressed genes in the liver, we noticed the presence of several genes encoding proteins involved in mRNA translation, including the components of the translation pre-initiation complex 5,6. In its inactive state, this complex is composed of the mRNA cap-binding protein eukaryotic translation initiation factor 4E (EIF4E) bound to the hypophosphorylated form of EIF4E-binding protein (4E-BP) that acts as a translational repressor. Upon stimulation, phosphorylation of 4E-BP releases EIF4E, which can then interact with the scaffold protein eIF4G and the rest of the EIF4F complex (EIF4A, EIF4B, and EIF4H) to initiate translation [7]. We therefore investigated whether the circadian clock might coordinate translation in mouse liver. Here we indeed show that the circadian clock controls the transcription of translation initiation factors as well as the rhythmic activation of signaling pathways involved in their regulation. As a consequence, the circadian clock influences the temporal translation of a subset of mRNAs mainly involved in ribosome biogenesis. In addition, the circadian oscillator regulates the transcription of ribosomal protein mRNAs and ribosomal RNAs. These results demonstrate for the first time the major role of the circadian clock in ribosome biogenesis.

## Results

### Rhythmic Expression and Activation of Components of the Translation Pre-initiation Complex

**Figure 1:**
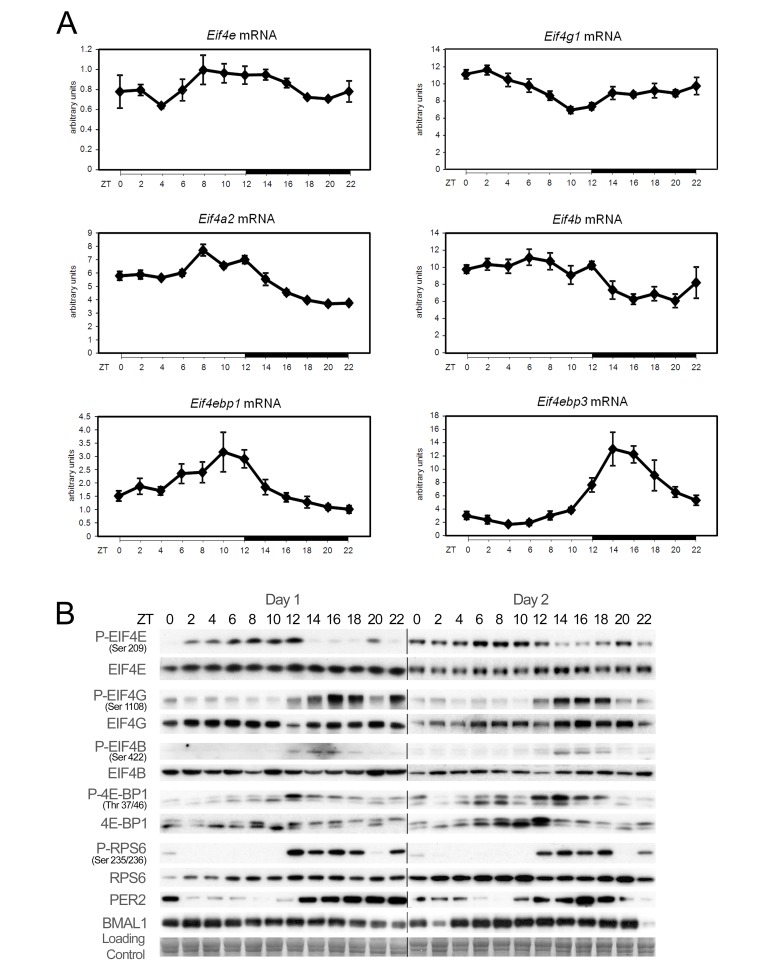
Temporal expression and phosphorylation of translation initiation factors. (A) Temporal mRNA expression profile of translation initiation factors in mouse liver. For each time point, data are mean ± standard error of the mean (SEM) obtained from four independent animals. (B) Temporal protein expression and phosphorylation of translation initiation factors in mouse liver during two consecutive days. Western blots were realized on total or nuclear (PER2 and BMAL1) liver extracts. PER2 and BMAL1 accumulations are shown as controls for diurnal synchronization of the animals. Naphtol blue black staining of the membranes was used as a loading control. The lines through gels indicate where the images have been cropped. The zeitgeber times (ZT), with ZT0, lights on; ZT12, lights off, at which the animals were sacrificed, are indicated on each panel.

We investigated whether the circadian clock might coordinate translation in mouse liver. Indeed, quantitative reverse transcription (RT)-PCR analyses confirmed that mRNAs of most of the factors involved in translation initiation are rhythmically expressed with a period of 24 h (Figure 1A; statistical analyses are given in Table S1). Interestingly, while we did not observe any significant variations in protein abundance, rhythmic phosphorylations were strongly manifested during two consecutive days, emphasizing the robustness of these rhythms (Figure 1B; quantification and statistical analyses of the data are given on Figure S1 and Table S2). EIF4E is mostly phosphorylated during the day, with a peak at the end of the light period (ZT6-12), whereas EIF4G, EIF4B, 4E-BP1, and ribosomal protein (RP) S6 (RPS6) are mainly phosphorylated during the night, which is, in the case of nocturnal animals like rodents, the period when the animals are active and consume food.

**Figure 2:**
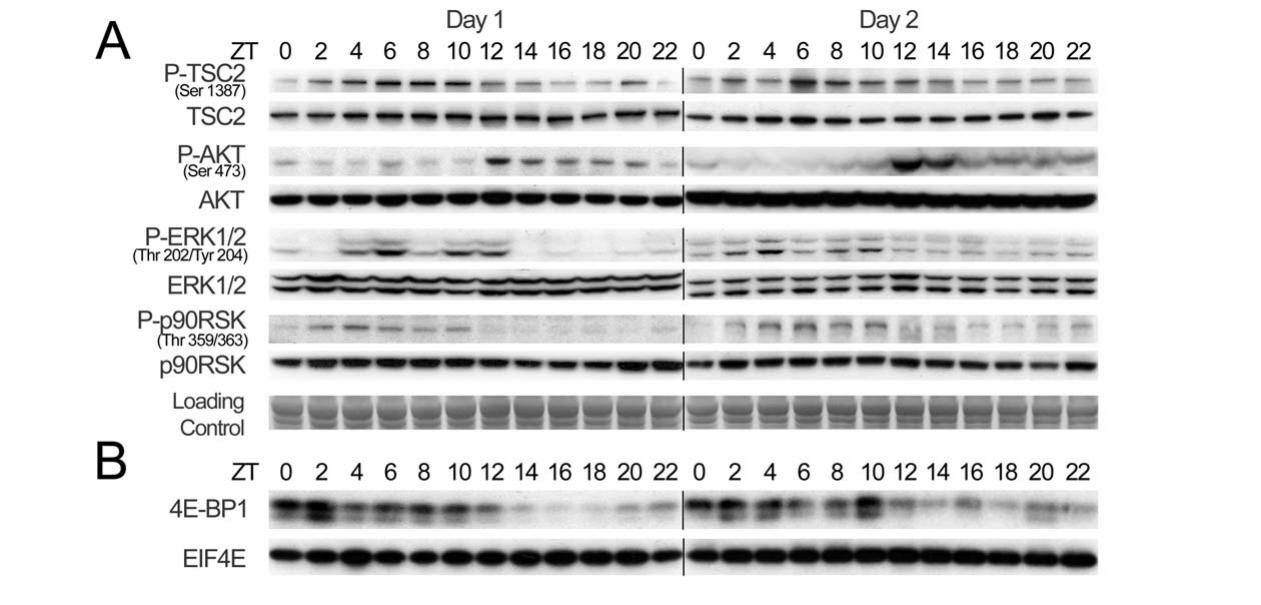
Temporal activation of signaling pathways controlling translation initiation. (A) Temporal expression and phosphorylation of representative proteins of key signaling pathways regulating translation initiation in mouse liver during two consecutive days. Western blots were performed on total liver extracts. Naphtol blue black staining of the membranes was used as a loading control. (B) Temporal binding of EIF4E and 4E-BP1 to 7-methyl-GTP-sepharose during two consecutive days. Total liver extracts were incubated with 7-methyl-GTP beads mimicking the mRNA cap structure. After washing of the beads, bound proteins were analyzed by Western blotting. The zeitgeber times (ZT), with ZT0, lights on; ZT12, lights off, at which the animals were sacrificed, are indicated on each panel. The lines through gels indicate where the images have been cropped.

Phosphorylation of these factors is well characterized and involves different signaling pathways [8] whose reported activity perfectly correlates with the observed phosphorylation rhythm. EIF4E is phosphorylated by the extracellular signal-regulated protein kinase (ERK)/mitogen-activated protein kinase (MAPK)-interacting kinase (MNK) pathway [9], which is most active during the day, at the time when EIF4E reaches its maximum phosphorylation (Figure 2A; quantification and statistical analyses of the data are given on Figure S2 and Table S2). On the other hand, EIF4G, EIF4B, 4E-BP1, and RPS6 are mainly phosphorylated by the target of rapamycin (TOR) complex 1 (TORC1) [10], which is activated during the night, at the time when the phosphorylation of these proteins reaches its maximum level. TORC1, in turn, is negatively regulated by the tuberous sclerosis protein complex (TSC), whose activity is under the control of the phosphoinositide 3-kinase (PI3K)/AKT, ERK, and the energy sensing 5′ adenosine monophosphate-activated protein kinase (AMPK) pathways [10],[11]. As reported [12], AMPK is active during the day and mediates the activation of TSC2, contributing to the repression of TORC1 in the period of energy and nutrient restriction. Conversely, during the night, TORC1 is activated probably through TSC2 inhibition by PI3K via TORC2 [13].

Interestingly, we found that *mTor*, its partner *Raptor*, as well as its regulating kinase *Map3k4*, are also rhythmically expressed, thus potentially further contributing to the rhythmic activation of TORC1 (Figure S3; Table S1). ERK is activated during the day in synchrony with the rhythmic expression of *Mnk2* (Figure S3), contributing to EIF4E phosphorylation during this period. However, its downstream target RPS6 Kinase (RSK) seems to contribute only marginally to the phosphorylation of RPS6 in mouse liver (Figures 1B and 2A). The rhythmic phosphorylation of 4E-BP1 resulted in its release from the mRNA cap-mimicking molecule 7-methyl-GTP from ZT14 to ZT22 (Figure 2B; Table S2), allowing the rhythmic assembly of the EIF4F and potentially mRNA translation.

The rhythmic expression of mRNA encoding translation initiation factors, TORC1 complex component, and a kinase activating these factors is independent of light as it is maintained under constant darkness, even if the phase seems to be advanced (Figure S4A). Interestingly, activation of the TORC1 pathway is also maintained under constant darkness but with an advanced phase (Figure S5A). Since nutrient availability is a potent activator of the TORC1 pathway [13], we asked whether these parameters are also rhythmic under conditions of starvation. We found that expression of mRNA encoding translation initiation factors, TORC1 complex component, and a kinase activating these factors is still rhythmic under starvation (Figure S4B), even when this starvation occurs under constant darkness (Figure S4C). This result unambiguously demonstrates the role of the circadian clock in the expression of these genes. In addition, phosphorylations of RPS6 and 4E-BP1 are still rhythmic under starvation, whether or not the mice are under a light-dark regimen or in constant darkness (Figure S5B and S5C), confirming previously published observations [14]. Interestingly, TORC1 activation is in opposite phase with the clock-dependent rhythmic activation of autophagy in mouse liver [15], a process inhibited by TORC1 but able to generate amino acids that can in turn activate TORC1 [16]. This might suggest that the circadian clock can regulate the two processes in a coordinated fashion. Importantly, rhythmic activation of TORC1 is not restricted to the liver as the same phosphorylation rhythm is found in kidney and heart, albeit with reduced amplitude (Figure S6). Meanwhile, TORC1 activation is constant in brain, lung, and small intestine, suggesting that the rhythmic nutrient availability due to the circadian clock-regulated feeding behavior is not sufficient by itself to explain the rhythmic activation of TORC1.

### Characterization of Rhythmically Translated mRNAs

**Figure 3:**
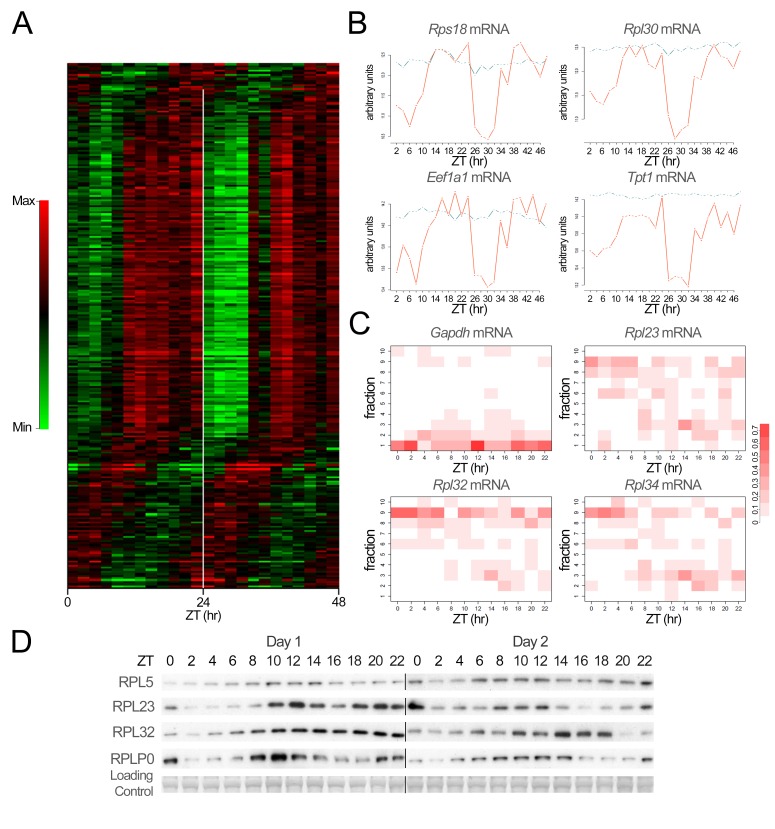
Rhythmic translation of ribosomal proteins in mouse liver. (A) Temporal expression profiles of microarray probes showing a rhythmic ratio of polysomal to total RNAs, ordered by phase. For visualization, data were mean centered and standardized. Log-ratios are color-coded so that red indicates high and green low relative levels of polysomal mRNAs compared to the total fraction. (B) Examples of temporal expression profiles of a subset of rhythmically translated 5′-TOP genes identified in our microarray experiment. Traces represent the levels of mRNA expression measured by microarray in the total RNA (blue line) and polysomal fraction (red line). Data are represented in log scale following standard normalization. (C) Temporal location of *Gapdh* and selected genes showing translational regulation mRNA on the different gradients obtained after polysomes purification. Pools of RNA obtained from four animals were used for each fraction at each time point. The color intensity represents for each time point the relative abundance of the mRNA in each fraction. Fractions 1–2 represent heavy polysomes, 2–3, light polysomes, and 9–10, free mRNAs. Note that even for *Gapdh* mRNA, translation slightly decreases at the end of the light period. (D) Temporal expression of selected rhythmically translated ribosomal proteins in liver cytoplasmic extracts during two consecutive days. Naphtol blue black staining of the membranes was used as a loading control. The lines through gels indicate where the images have been cropped. The zeitgeber times (ZT) at which the animals were sacrificed are indicated on each panel.

Diurnal binding of 4E-BP to EIF4E suggested that translation might be rhythmic in the liver. To test this hypothesis and to identify potential rhythmically translated genes, we purified polysomal RNAs, a RNA sub-fraction composed mainly of actively translated mRNA, every 2 h during a period of 48 h. We found that relative amount of this polysomal fraction follows a diurnal cycle, showing that a rhythmic translation does occur in mouse liver (Figure S7). This result confirms original observations based on electron microscopy and biochemical studies [17],[18]. We therefore decided to characterize these rhythmically translated mRNAs through comparative microarray analysis of polysomal and total RNAs. While the obtained profiles in polysomal and total RNAs fractions are highly similar for most mRNAs (examples of rhythmic mRNAs are given on Figure S8), 249 probes showed a non-uniform ratio in diurnal polysomal over total mRNAs (Figure 3A). This means that approximately 2% of the expressed genes are translated with a rhythm that is not explained by rhythmic mRNA abundance as in most cases, the total mRNA levels were constant while the polysomes-bound mRNA levels fluctuated during the 24-h cycle (Figures 3B and S9). Among translationally regulated genes, 70% were found in the polysomal fraction during the same time interval, starting at ZT8 before the onset of the feeding period and finishing at the end of the dark period (Tables S3 and S4). Most of these genes belonged to the 5′-terminal oligopyrimidine tract (5′-TOP) family, known to be regulated by TORC1 [19], but also by the level and phosphorylation state of EIF4E [20],[21]. 5′-TOP genes are themselves involved in translation via ribosome biogenesis and translation elongation (Table S4).

After confirmations of these results by quantitative RT-PCR (Figure S10), we wished to validate the periodicity in the amount of mRNAs purified in the different fractions obtain during polysomes purification over a 24-h period. Whereas a constitutively translated mRNA such as *Gapdh* is found all the time in the polysomal fraction (with a small decrease in the middle of the light period when overall translation decreases), mRNAs coding for RPs are associated with the polysomal fraction only starting towards the end of the light period (ZT8) and during the dark period (Figure 3C). This result demonstrates a dynamic translation initiation of 5′-TOP mRNA starting before the onset of the feeding period, with a maximum at the beginning of the dark period.

Next, we wanted to confirm that this rhythmic translation had an impact on the protein levels. With respect to RPs, while the half-life of mature ribosomes is approximately 5 d in rodent liver [22], newly synthesized RPs have a half-life of only a few hours, as most of them are rapidly degraded after translation during the ribosome assembly process in the nucleolus [23]. We thus expected a rhythmic expression of this subpopulation of newly synthesized RPs in the soluble cytosolic fraction depleted of ribosomes after sedimentation. Indeed, under these conditions, RPs show a rhythmic abundance with highest expression during the night (Figure 3D; quantification and statistical analyses of the data are given on Figure S11 and Table S2). In some cases, we noticed a shallow decrease at ZT16-18, potentially reflecting transport of RPs into the nucleolus for ribosome assembly.

### Control of RPs mRNA and rRNA Transcription by the Circadian Clock

**Figure 4:**
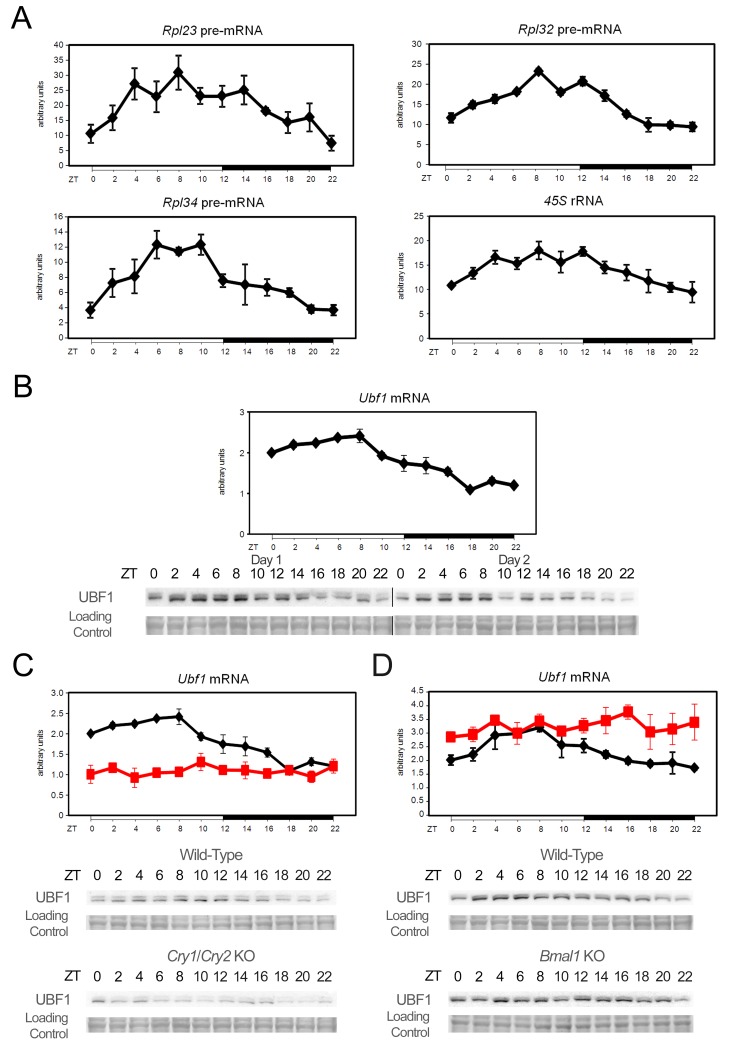
Rhythmic transcription of RP mRNA and rRNA through circadian clock regulated expression of UBF1. (A) Temporal real-time RT-PCR profile of RP pre-mRNA and 45S rRNA precursor expression in mouse liver. For each time point, data are mean ± standard error of the mean (SEM) obtained from four independent animals. (B) Temporal *Ubf1* mRNA (upper panel) and protein (lower panel) expression in mouse liver. mRNA were measured by real-time RT-PCR and, for each time point, data are mean ± SEM obtained from four independent animals. UBF1 protein expression was measured by Western blot on nuclear extracts during two consecutive days. The lines through gels indicate where the images have been cropped. (C–D) Temporal *Ubf1* expression in mice devoid of a functional circadian clock. *Ubf1* expression was measured by real-time RT-PCR with liver RNAs obtained from arrhythmic *Cry1*/*Cry2* (C) and *Bmal1* (D) KO mice and their control littermates (upper panel). Data are mean ± SEM obtained from three and two animals, respectively. Black line corresponds to the WT animals and red line to the KO. Protein levels (lower panel) were measured by Western blot on nuclear extracts. The zeitgeber times (ZT) at which the animals were sacrificed are indicated on each panel. Naphtol blue black staining of the membranes was used as a loading control.

 In addition to translational regulation, we also observed a diurnal expression of RP mRNAs, albeit with a small average peak to trough amplitude of approximately 1.2. Taking into account their relatively long half-life (11 h) [24], we hypothesized that this minor fluctuation might reflect more pronounced rhythmic amplitudes in transcription as amplitude decreases with half-life [25]. In addition, it has recently been shown that the transcription of several RP mRNAs is directly controlled by the molecular oscillator in *Drosophila* head [26]. Indeed, pre-mRNA accumulation of several RP exhibited a rhythmic transcription, with an average amplitude of 3.5-fold with a maximum at ZT8, just before the activation of their translation (Figure 4A; statistical analyses are given in Table S1). In addition, we found that the synthesis of the ribosome constituent precursor 45S rRNA is also rhythmic and synchronized with RP mRNAs transcription, indicating that all elements involved in ribosome biogenesis are transcribed in concert, then translated or matured. In yeast [27] and *Drosophila* [28], transcription of RP mRNAs appears to be coordinated with rRNA transcription, which is a rate limiting step in ribosome biogenesis. On the other hand, in mammals, rRNA transcription is highly regulated by the upstream binding factor (UBF), which establishes and maintains an active chromatin state [29]. Remarkably, we found that UBF1 is rhythmically expressed in mouse liver at both mRNA and protein levels (Figure 4B; quantification and statistical analyses of the data are given in Figure S12A and Tables S1 and S2), in phase with RP mRNAs and rRNAs transcription. In addition, rhythmic transcription of *Ubf1* and *Rpl23* genes is also independent of light and food (Figure S4).

**Figure 5:**
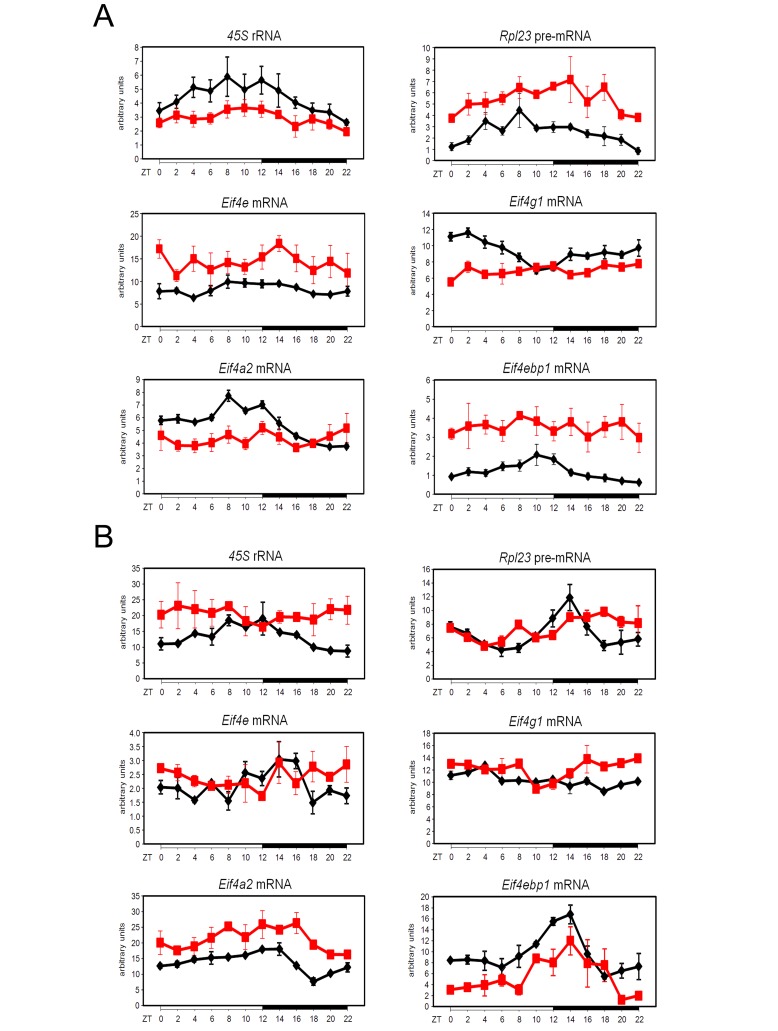
Rhythmic RNA expression of factors involved in ribosomes biogenesis is disrupted in arrhythmic *Cry1*/*Cry2* and *Bmal1* KO mice. Temporal expression of factors involved in ribosomes biogenesis in *Cry1*/*Cry2* (A) and *Bmal1* (B) KO mice and their control littermates. Temporal real-time RT-PCR expression profile of 45S rRNA precursor, *Rpl23* pre-mRNA, and translation initiation factors expression in mouse liver. Black line corresponds to the WT animals and red line to the KO. For each time point, data are mean ± SEM obtained from three (A) and two (B) independent animals. The zeitgeber times (ZT) at which the animals were sacrificed are indicated on each panel.

To test whether *Ubf1* transcription is regulated by the circadian clock, we characterized its expression in arrhythmic *Cry1*/*Cry2* knockout (KO) [30] and *Bmal1* KO [31] mice, which are devoid of a functional circadian clock. Indeed, these mice do exhibit an arrhythmic pattern of activity under constant darkness, which is in general correlated with an arrhythmic feeding behaviour. As TORC1, as well as other signaling pathways, are in part regulated by feeding through nutrient availability, we expect a temporally discontinuous and erratic activation of these pathways in the KO mice under unrestricted feeding. To verify this hypothesis, we measured activation of the TORC1, AKT, and ERK pathways in *Cry1*/*Cry2* and *Bmal1* KO kept in constant darkness. As shown in Figure S13A, the rhythmic activation of these signaling pathways is indeed lost under this condition, confirming their arrhythmic activation. To highlight the role of the feeding regimen on this activation, we kept *Cry1*/*Cry2* KO mice in constant darkness and sacrificed them at CT12. We found a strong inter-individual variability in the activation of the TORC1, AKT, and ERK pathways, reflecting the arrhythmic feeding rhythm of these animals (Figure S13B). To circumvent this caveat and study the rhythmic translation in mice devoid of a functional molecular oscillator, we decided to place *Cry1*/*Cry2* and *Bmal1* KO under a light-dark regimen to keep a normal diurnal feeding behaviour due to masking. In addition, mice had access to food only during the dark phase to eliminate the effect of a potential disturbed feeding behaviour. Under these conditions, KO mice had a rhythmic feeding behaviour and thus potential differences in protein levels or pathway activity cannot be attributed to the arrhythmic feeding behaviour of these animals. We indeed found that UBF1 rhythmic expression is dependent on a functional circadian clock as it is impaired in both animal models (Figure 4C and 4D; quantification and statistical analyses of the data are given in Figure S12B and Tables S5, S6, S7, S8). However, if UBF1 expression is persistently low in *Cry1*/*Cry2* KO mice, this expression is constantly high in *Bmal1* KO mice, suggesting the control of *Ubf1* by a circadian clock-regulated transcription repressor. In addition, we observed that these animals lose also the synchrony and coordination of 45S rRNA and RP pre-mRNAs transcription (Figures 5, S14, and S15; statistical analyses of the data are given in Table S5 and S6). Indeed, decreased UBF1 expression in *Cry1*/*Cry2* KO mice is correlated with lower 45S rRNA transcription, but higher and delayed RP pre-mRNAs transcription. Interestingly, *Bmal1* KO mice present a complete arrhythmic transcription of RP pre-mRNAs, highlighting the crucial role of the circadian clock in the coordination of rRNA and RP mRNAs transcription.

### The Circadian Clock Controls Expression and Activation of Components of the Translation Initiation Complex

**Figure 6:**
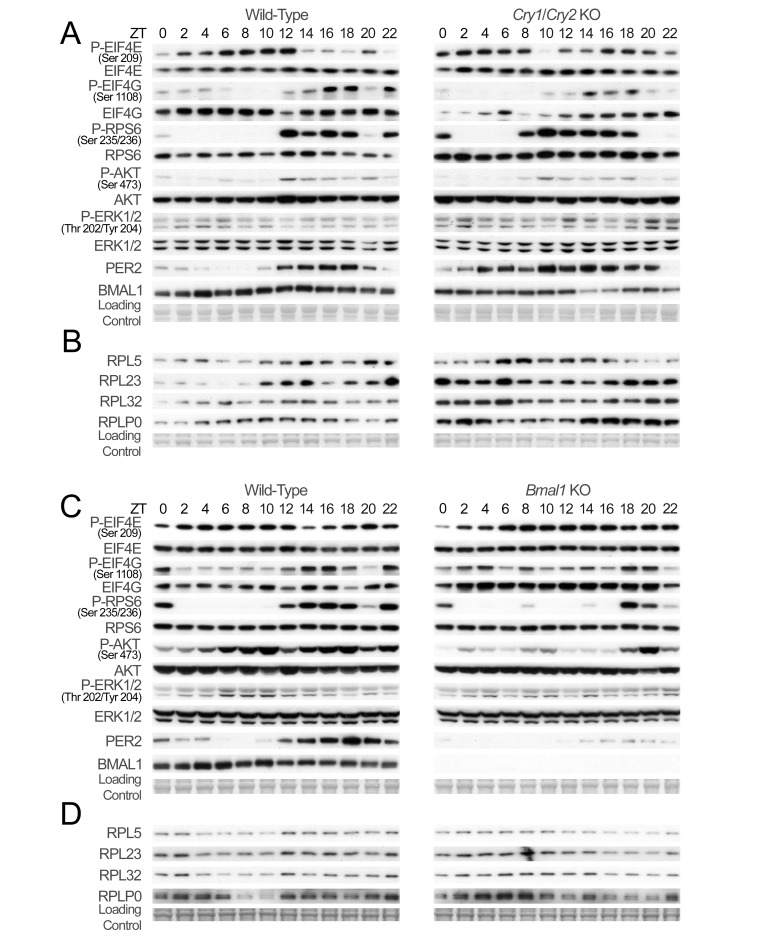
Rhythmic expression and phosphorylation of actors of ribosomes biogenesis is disrupted in arrhythmic *Cry1*/*Cry2* and *Bmal1* KO mice. (A–C) Temporal expression and phosphorylation of translation initiation factors and representative indicators of signaling pathways controlling their activation in *Cry1*/*Cry2* (A) and *Bmal1* (C) KO mice and their control littermates. Western blots were realized on total or nuclear (PER2 and BMAL1) liver extracts from WT (left panel) and KO (right panel) animals. (B–D) Temporal expression of selected rhythmically translated ribosomal proteins in liver from *Cry1*/*Cry2* (B) and *Bmal1* (D) KO mice and their control littermates. Western blots were realized on cytoplasmic extracts from WT (left panel) and KO (right panel) animals. The zeitgeber times (ZT) at which the animals were sacrificed are indicated on each panel. PER2 and BMAL1 accumulations are shown as controls for diurnal synchronization of the animals. Naphtol blue black staining of the membranes was used as a loading control.

Rhythmic expression of genes coding for components of the translation initiation complex is strongly dampened or phase-shifted in both KO models, in addition to an altered level of expression (Figures 5, S14, and S15; statistical analyses of the data are given in Tables S5 and S6). However, we did not observe in general any significant variations in protein abundance, excepting a slight increase in EIF4E expression in *Cry1*/*Cry2* KO mice, reflecting increased mRNA expression (Figure 6A and 6C; quantification and statistical analyses of the data are given in Figures S16, S17; Tables S7 and S8). The variations in EIF4G levels reflect more the changes in its phosphorylation state, which regulates its stability [32]. While most of the signaling pathways are still rhythmic in *Cry1*/*Cry2* KO mice, except for the ERK pathway and the downstream phosphorylation of EIF4E, which loses its rhythmic activation, the phase of the activation of the TORC1 and AKT pathways are advanced in comparison to wild-type (WT) mice (Figures 6A and S16; quantification and statistical analyses of the data are given in Table S7). As a consequence, the rhythmic expression of RPs is altered in *Cry1*/*Cry2* KO mice (Figure 6B; quantification and statistical analyses of the data are given in Table S7), with an increased level of expression, likely because of the increased RP pre-mRNAs and EIF4E levels [20], and a delayed phase of expression. Most of the rhythmic activation of the three pathways is also strongly altered in *Bmal1* KO mice (Figures 6C and S17; quantification and statistical analyses of the data are given in Table S8). As shown in Figure 6D, the phase of RPs rhythmic expression is severely advanced with a maximum of expression in the middle of the day instead of the night (Figure 6D; quantification and statistical analyses of the data are given in Table S8).

## Discussion

### Regulation of Ribosome Biogenesis by the Circadian Clock

**Figure 7:**
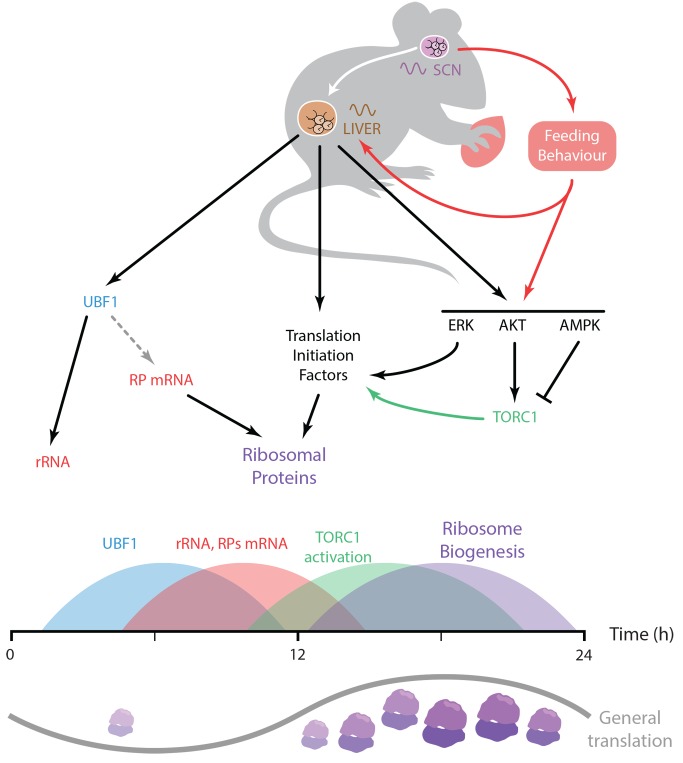
Model describing the coordination of ribosome biogenesis by the circadian clock. The molecular oscillator in the master circadian pacemaker localized in the SCN of the hypothalamus synchronizes peripheral clocks, including liver clock, and, in parallel, regulates feeding behavior, which itself influences peripheral oscillator. The liver circadian clock controls expression of translation initiation factors, and rRNA, and conceivably RP mRNA, through regulation of UBF1. In addition, in association with signals from nutrients, the molecular clock, via the TORC1 pathway, coordinates the rhythmic activation of signaling pathways controlling translation of RP and, in turn, ribosome biogenesis. This succession of events coordinated by the circadian clock finally leads to a subtle rhythmic change of general translation in mouse liver.

The results presented here show that the molecular circadian clock controls ribosome biogenesis through the coordination of transcriptional, translational, and post-translational regulations. Moreover, the data strongly suggest that a functional molecular oscillator is required for a timely coordinated transcription of translation initiation factors, RP mRNAs, and rRNAs. The clock modulates the rhythmic activation of signaling pathways controlling translation through the TORC1 pathway, translation of RPs, and ribosome biogenesis (Figure 7). Interestingly, it has been reported that the size of the nucleolus, the site of rRNA transcription and ribosome assembly, follows a diurnal pattern with a maximum in the middle of the dark period [33], which thus occurs in synchrony with the observed accumulation of RPs in the liver. The observed rhythmic ribosome biogenesis is substantiated by the previous observation showing that both size and organization of the nucleolus are directly related to ribosome production [34].

Remarkably, a coordinated rhythmic regulation of transcriptional and translational events for the biogenesis of ribosomes has also been suggested for the filamentous fungus *Neurospora crassa* [35] and for plants [36],[37]. Since ribosome biogenesis is one of the major energy consuming process in cells [38], its tight control is primordial to reduce interferences with other biological processes. In the case of mouse liver, we estimate that the decrease of translation during the light period is equivalent to 20% of the total translation (Figure S7), in agreement with previously published results [17]. Although moderate, this decrease affects translation of housekeeping genes like *Gapdh* (Figure 3C) and probably the translation of other genes. It means that the increase in ribosome biogenesis during the night could potentially influence the translation of many other mRNAs, however with a magnitude sufficiently low to not allow its detection by our method.

Nevertheless, it is clear that this energy-consuming process has to be confined to a time when energy and nutrients are available in sufficient amount, which, in the case of rodents, is during the night period when the animals are active and consume food. Hence, all the elements required for translation have to be ready to start ribosome biogenesis during that time. This is achieved by increasing levels of rRNAs and RP pre-mRNAs just before the onset of the night, synchronized with the phosphorylation of EIF4E that increases 5′-TOP mRNAs translation [21]. Activation of the TORC1 pathway during this period promotes RPs synthesis, rRNAs maturation, and ribosome assembly. In addition activation of the ERK pathway correlates also with ribosome biogenesis [39], strengthening the rhythmic nature of this process. Accordingly, orchestration of ribosome biogenesis by the circadian clock represents a nice example of anticipation of an obligatory gated process through a complex organization of transcriptional, translational, and post-translational events.

### Coordination of Rhythmic Activation of Cellular Signaling Pathways by the Circadian Clock

As described in the introduction, the mammalian molecular circadian oscillator consists in interlocked feedback loops of transcription factors that generate a complex network of rhythmically expressed genes [3]. Within the core molecular clock, increasing evidence shows that post-translational modifications play a crucial role in the generation of circadian rhythms [40]. However, the circadian clock is also able to coordinate rhythmic post-translational activation of signaling pathways not directly involved in the molecular oscillator but rather in the sensing of the environment. The first described example consisted in the rhythmic activation of ERK in the suprachiasmatic nucleus (SCN) of the hypothalamus where the master circadian pacemaker is localized: if light stimulates ERK phosphorylation in the SCN in a time-dependent fashion, circadian ERK phosphorylation continues also in constant darkness, suggesting a crucial role of the circadian clock in this process [41]. Interestingly, the same observations have been made for the TORC1 pathway in the SCN [42],[43], and for the PI3K/AKT pathway in the retina [44]. Considering the fact that these two pathways have been recently identified as a potent regulators of circadian activity in *Drosophila* [45], we expect that the role of the circadian clock-coordinated signaling pathways on circadian physiology will probably be emphasized in other organisms in the near future.

With respect to rhythmic activation of signaling pathways in the liver, there are only few examples of such regulations. One example is the rhythmic activation of the PI3K/AKT pathway that is associated with food metabolism and rhythmic feeding behavior [46]. Recently, we also described a circadian clock-dependent rhythmic activation of the unfolded protein response regulating liver lipid metabolism [47]. In addition, it has been shown that the circadian clock is also able to regulate autophagy in mouse liver [15]. In this context, our discovery of the rhythmic ribosome biogenesis through coordination of the rhythmic activation of signaling pathways constitutes an important new element in this area of research.

### Translation, Circadian Clock, and Longevity

It has long been known that caloric restriction or intermittent fasting increases lifespan in a wide variety of models [48]. Increased lifespan has also been linked to the reduced activation of the TORC1 pathway, which, in turn, provokes a reduced mRNA translation [49],[50]. The role of the TORC1 pathway in this translation-dependent extension of lifespan has been genetically confirmed in *Caenorhabditis elegans* [51] and *Drosophila* [52],[53]. A similar scenario is also considered in mice since treatment with the TOR inhibitor rapamycin [54] or deletion of the TORC1 downstream protein kinase S6K1 [55] lead to increased lifespan. In addition, downregulation of various components of the EIF4F complex extends lifespan in *C. elegans* [56]–[59], whereas inhibition of RPs genes expression extends lifespan in both *Saccharomyces cerevisiae* [60] and *C. elegans* [56]. Hence, keeping ribosome biogenesis, and translation in general, to their minimum levels plays a major role in the regulation of longevity [61]. Interestingly, all the genetically modified animal models presenting a disrupted circadian clock [62]–[64] or mice subjected to chronic jet lag [65] are subjected to premature aging and reduced lifespan. The deregulation of many other circadian-clock regulated processes can reduce life expectancy, like reduced xenobiotic detoxification [66]. We thus believe that the potential role of disorganized ribosome biogenesis on life expectancy, observed in animals devoid of a circadian clock, will be an exciting subject for further studies.

## Material and Methods

### Animal Experiments

All animal studies were conducted in accordance with our regional committee for ethics in animal experimentation and the regulations of the veterinary office of the Canton of Vaud. C57Bl/6J mice were purchased from Janvier (Le Genest) or Charles River Laboratory (L'Arbresle). *Bmal1* floxed mice have been previously described [67]. These mice were crossed with mice expressing the CRE recombinase under the control of the CMV promoter [68] to obtain *Bmal1* KO mice. *Cry1*/*Cry2* double KO mice [30] in the C57Bl/6J genetic background have been previously described [69]. In all experiments, male mice between 10 and 12 wk of age are used. Unless noted otherwise, mice were maintained under standard animal housing conditions, with free access to food and water and in 12-h light/12-h dark cycles. However, for all experiments, animals were fed only at night during 4 d before the experiment to reduce effects of feeding rhythm. For experiments in constant darkness, mice were shifted into complete darkness after the last dark period and then sacrificed every 2 or 4 h during the next 48 h. For starvation experiments, mice were deprived from food during one complete night and then during the following 24 h, mice were sacrificed every 2 or 4 h.

### Polysome Purification

Livers were homogenized in lysis buffer containing 20 mM HEPES (pH 7.6), 250 mM NaCl, 10 mM MgCl_2_, 10 mM DTT, 20 µg/ml cycloheximid, 10 U/µl RNase inhibitor, and a protease inhibitor cocktail containing 0.5 mM PMSF, 10 µg/ml Aprotinin, 0.7 µg/ml Pepstatin A, and 0.7 µg/ml Leupeptin. The homogenates were centrifuged 10 min at 9,500 *g* and 1 mg/ml heparin, 0.5% Na deoxycholate, and 0.5% Triton ×100 were added to the supernatant. 50 mg of lysate were deposited on a 36 ml 7% to 47% sucrose gradient in a buffer containing 20 mM HEPES (pH 7.6), 100 mM KCl, 5 mM MgCl_2_, and 1 mM DTT. After 4 h 30 min of centrifugation at 130,000 *g* and 4°C, the gradient was divided in fractions of approximately 1 ml with a peristaltic pump. Optic density of the fractions at 260 nm was measured to establish the polysomal profile in the gradient. Fractions were finally pooled in ten fractions. An example of polysome profile is given on Figure S18. RNAs were then extracted according to the protocol described by Clancy et al. [70] that we slightly modified. Briefly, fractions were precipitated by the addition of three volumes of ethanol and kept overnight at −80°C. After 30 min of centrifugation at 5,200 *g*, RNAs were extracted from the non-soluble fraction by classical protocol [71].

### RNA Extraction and Analysis

Liver RNAs were extracted and analysed by real-time quantitative RT-PCR, mostly as previously described [25]. Briefly, 0.5 µg of liver RNA was reverse transcribed using random hexamers and SuperScript II reverse transcriptase (Life Technologies). The cDNAs equivalent to 20 ng of RNA were PCR amplified in triplicate in an ABI PRISM 7700 Sequence Detection System (Applied Biosystem) using the TaqMan or the SYBR Green technologies. References and sequences of the probes are given in Tables S9 and S10, respectively. *Gapdh* mRNA (total RNA) or 28S rRNA (polysomal RNA) were used as controls.

### Microarray Experiments

Liver polysomal and total RNAs were extracted independently from two mice sacrificed every 2 h during 48 h. For polysomal RNAs, we pooled fractions 1 and 2 from the ten fractions obtained during the extraction and containing heavy polysomes. 3 µg of polysomal and total RNAs from each animal from each time point were pooled. These 6 µg of polysomal and total RNAs were used for the synthesis of biotinylated cRNAs according to Affymetrix protocol, and hybridized to mouse Affymetrix Mouse Genome 430 2.0 arrays. The chips were washed and scanned, and the fluorescence signal analysed with Affymetrix software. Data are deposited on the Gene Expression Omnibus database under the reference GSE33726 (http://www.ncbi.nlm.nih.gov/geo/query/acc.cgi?token=rpwvtoqogkamwrm&acc=GSE33726).

The raw data of all 48 arrays were normalized together using the robust multiarray average (RMA) method [72]. For the analysis, we filtered out all probesets corresponding to introns using the Ensembl annotation and then only kept genes with a sufficient expression level (we kept genes whose probe signal in the total fraction was above 5 in log2 scale). For the identification of circadian probesets, the 24-h Fourier component (F24) and the phase were computed using established methods [73]. The associated *p*-value (*p*) was calculated using the Fisher test (*p* = (1−*s*)^10^) [73]. For the identification of rhythmically translated genes, the difference between polysomal and total RNAs was subjected to Fourier analysis and we selected probesets giving a *p*-value inferior to 0.001. In addition, we requested that the peak to trough amplitude in the polysomal signal be above 1.2-fold.

### Nuclear and Cytoplasmic Protein Extractions and Analysis

Nuclear and cytoplasmic proteins were extracted mostly as described [25]. Briefly, liver were homogenized in sucrose homogenization buffer containing 2.2 M sucrose, 15 mM KCl, 2 mM EDTA, 10 mM HEPES (pH 7.6), 0.15 mM spermin, 0.5 mM spermidin, 1 mM DTT, and the same protease inhibitor cocktail as for polysomes extraction. Lysates were deposited on a sucrose cushion containing 2.05 M sucrose, 10% glycerol, 15 mM KCl, 2 mM EDTA, 10 mM HEPES (pH 7.6), 0.15 mM spermin, 0.5 mM spermidin, 1 mM DTT, and a protease inhibitor cocktail. Tubes were centrifuged during 45 min at 105,000 *g* at 4°C. After ultra-centrifugation, supernatants containing soluble cytoplasmic proteins were harvested, homogenised, and centrifuged for 2 h at 200,000 *g* to remove ribosomes. These supernatants constitute cytoplasmic extracts. The nucleus pellets were suspended in a nucleus lysis buffer composed of 10 mM HEPES (pH 7.6), 100 mM KCl, 0.1 mM EDTA, 10% Glycerol, 0.15 mM spermine, 0.5 mM spermidine, 0.1 mM NaF, 0.1 mM sodium orthovanadate, 0.1 mM ZnSO4, 1 mM DTT, and the previously described protease inhibitor cocktail. Nuclear extracts were obtained by the addition of an equal volume of NUN buffer composed of 2 M urea, 2% nonidet P-40, 600 mM NaCl, 50 mM HEPES (pH 7.6), 1 mM DTT, and a cocktail of protease inhibitor, and incubation 20 min on ice. After centrifugation during 10 min at 21,000 *g*, the supernatants were harvested and constitute nuclear extracts.

25 µg of nuclear or 12.5 µg cytoplasmic extracts were used for western blotting. After migration, proteins were transferred to PVDF membranes and Western blotting was realized according to standard procedures. References for the antibodies are given in Table S11.

### Total Protein Extraction and Analysis

Organs were homogenized in lysis buffer containing 20 mM HEPES (pH 7.6), 100 mM KCl, 0.1 mM EDTA, 1 mM NaF, 1 mM sodium orthovanadate, 1% Triton X-100, 0.5% Nonidet P-40, 0.15 mM spermin, 0.5 mM spermidin, 1 mM DTT, and a protease inhibitor cocktail. After incubation 30 min on ice, extracts were centrifuged 10 min at 21,000 *g* and the supernatants were harvested to obtain total extracts.

65 µg of extract was used for Western blotting. After migration, proteins were transferred to PVDF membranes and Western blotting was realized according to standard procedures. References for the antibodies are given in Table S11.

### 7-methyl GTP Sepharose Affinity Protein Purification

7-methyl GTP sepharose 4B beads (GE Healthcare) were washed twice in the previously described liver lysis buffer. 250 µg of liver protein extracts were diluted in 500 µl of lysis buffer containing 1 mM DTT and a cocktail of protease inhibitor and incubated for 2 h on a rotating wheel at 4°C with 20 µl of beads. After incubation, cap-binding-proteins coated beads were washed five times in 500 µl of liver lysis buffer containing 0.5 mM PMSF and 1 mM DTT. 7-methyl GTP bound proteins were eluted by SDS-PAGE loading buffer, separated by SDS-PAGE, transferred to PVDF membranes, and analysed by Western blotting as described.

### Statistical Analysis of Genes and Proteins Expression

Mean and standard error of the mean were computed for each time point. The rhythmic characteristics of the expression of each gene or protein were assessed by a Cosinor analysis [74]. This method characterizes a rhythm by the parameters of the fitted cosine function best approximating the data. A period of 24 h was a priori considered. The rhythm characteristics estimated by this linear least squares method include the mesor (rhythm-adjusted mean), the double amplitude (difference between minimum and maximum of fitted cosine function), and the acrophase (time of maximum in fitted cosine function). A rhythm was detected if the null hypothesis was rejected with *p*<0.05. In such a case, the 95% confidence limits of each parameter were computed. The Cosinor 2.3 software used in this study has been elaborated by the Circadian Rhythm Laboratory at University of South Carolina and is freely available at this address: http://www.circadian.org/softwar.html. The statistical significance of differences in the mesor was evaluated by a Student's *t*-test.

## Supporting Information

Figure S1 **Temporal expression and phosphorylation of translation initiation factors in WT mice.** Mean ± standard error of the mean (SEM) (*n* = 3) densitometric values of the Western blot data depicted in Figure 1B were represented according to the zeitgeber time. Statistical analysis of these data is given in Table S2.
(TIF)

Figure S2 **Temporal expression and phosphorylation of proteins involved in signaling pathways activation and translational initiation in WT mice.** (A) Mean ± standard error of the mean (SEM) (*n* = 3) densitometric values of the Western blot data depicted in Figure 2A were represented according to the zeitgeber time. (B) Mean ± SEM (*n* = 2) densitometric values of the Western blot data depicted in Figure 2B were represented according to the zeitgeber time. Statistical analysis of these data is given in Table S2.
(TIF)

Figure S3 **Temporal expression of TORC1 components and of kinases regulating TORC1 and EIF4E activities in WT mice.** (A) Temporal expression of the TORC1 components *mTor* and *Raptor* at the mRNA level (upper panel) and protein level (lower panel) in mouse liver. mRNA expressions were measured by real-time RT-PCR. For each time point, data are mean ± standard error of the mean (SEM) obtained from four independent animals. Expression of mTOR and RAPTOR and its phosphorylation on Serine 792 were measured by Western blot on total extracts. The phosphorylation of RAPTOR on Serine 792 by AMPK has been shown to reduce TORC1 activity [75] and contributes to the inhibition of TORC1 during the day. Naphtol blue black staining of the membranes was used as a loading control. (B) Temporal expression of *Map4k3* (left panel) and *Mnk2* mRNA (right panel) in mouse liver. mRNA expressions were measured by real-time RT-PCR. For each time point, data are mean ± SEM obtained from four independent animals. MAP4K3 plays a role in the activation of TORC1 by amino acids [76], whereas MNK2 is involved in the ERK signaling cascade leading to the phosphorylation of EIF4E, which can play a role in 5′-TOP mRNA translation [9].
(TIF)

Figure S4 **Rhythmic expression of mRNA encoding translation initiation factors (** ***Eif4b*** **,** ***Eif4ebp3*** **), the TORC1 complex component** ***mTor*** **, the kinase activating these factors** ***Mnk2*** **, and proteins involved in rRNA synthesis (** ***Ubf1*** **) and ribosome biogenesis (** ***Rpl23*** **) is independent of food and light.** (A) Temporal expression in constant darkness. (B) Temporal expression during starvation. (C) Temporal expression during starvation in constant darkness. mRNA expressions were measured by real-time RT-PCR. For each time point, data are mean ± SEM obtained from three independent animals. The circadian (CT) or zeitgeber (ZT) times at which the animals were sacrificed are indicated on the bottom of the figures.
(TIF)

Figure S5 **Rhythmic activation of TORC1 still occurs in constant conditions.** (A) Temporal phosphorylation of TORC1 substrates during 48 h in constant darkness. The lines through gels indicate where the images have been cropped. (B) Temporal phosphorylation of TORC1 substrates during starvation. As reported [14], the period of activation seems to be shorter in these conditions. Interestingly, this activation is antiphasic with the rhythmic activation of autophagy in mouse liver [15], a process inhibited by TORC1 but able to generate amino acids that can in turn activate TORC1 [16]. (C) Temporal phosphorylation of the TORC1 substrate RPS6 during starvation in constant darkness. Temporal expression and phosphorylation of RPS6 and 4E-BP1 were measured by Western blot on total extracts. Naphtol blue black staining of the membranes was used as a loading control.The circadian (CT) or zeitgeber (ZT) times at which the animals were sacrificed are indicated on the top of the figures.
(TIF)

Figure S6 **Rhythmic activation of TORC1 in different mouse organs.** Temporal activation of the TORC1 pathway in mouse organs, revealed by phosphorylation of RPS6. As in the liver, this rhythmic activation is kept in kidney and heart, nevertheless with reduced amplitude (indicated by the blot with a shortest exposure). However, TORC1 activation is constant in brain, lung, and small intestine, suggesting that nutriment availability due to rhythmic feeding is not sufficient to explain this phenomenon. The zeitgeber times (ZT) at which the animals were sacrificed are indicated on each panel. Naphtol blue black staining of the membranes was used as a loading control.
(TIF)

Figure S7 **The polysomal fraction is rhythmic in mouse liver.** Temporal fraction of ribosomes in the polysomal fraction. The percentage is obtained by dividing the optical density obtained for the polysomal fraction by the total of optical density obtained for polysomes and monosomes (*n* = 5). The rhythmic nature of this fraction (and thus translation) is confirmed by cosinor analysis (*p*≤0.005, F[2,9] = 11.00, robustness = 61.3%, Mesor = 76.24, amplitude = 5.50, and phase = 18.09 h). This result confirms past biochemical [17] and morphometric [18] studies describing a rhythmic polysomal fraction in rodent liver with a nadir at ZT6. Interestingly, this time corresponds to the maximum of activity of AMPK [12], which inhibits TORC1 activity through phosphorylation of TSC2 [77] and RAPTOR [75]. The zeitgeber times (ZT) at which the animals were sacrificed are indicated on the bottom of the figure.
(TIF)

Figure S8 **The temporal profiles of polysomal mRNAs closely follow that of total mRNAs for most circadian genes, as exemplified by the** ***Period*** **genes.** (A) Temporal profiles ordered by phase in total (left panel) and polysomal RNA (right panel) fractions of microarray probes presenting a rhythmic profile in total mRNA fraction. Data were mean centered and standardized. Log-ratios are color-coded so that red indicates high and green low relative levels of mRNA. For most of the probes, the profiles are strikingly similar in the two fractions, indicating constant translational efficacy along the day. (B) Temporal expression of *Per1* (left panel) and *Per2* (right panel) mRNAs in polysomal (red line) and total (blue line) RNA fractions. Data are represented in log scale without any additional normalization than the one provided by the Affymetrix software. Although a regulation of PER1 expression at the translational level has been proposed [78],[79], this hypothesis is not confirmed by our in vivo data as the two profiles are extremely similar.
(TIF)

Figure S9 **Comparative diurnal expression profile of RNA in total and polysomal fractions.** Temporal profiles of total RNA (left panel) and polysomal RNA (right panel) fractions of microarray probes presenting a rhythmic polysomal/total RNA ratio. The profiles are ordered by the phase of the polysomal/total ratio phase. Data were mean centered and standardized. Log-ratios are color-coded so that red indicates high and green low relative levels of mRNA.
(TIF)

Figure S10 **Diurnal expression of selected 5′-TOP mRNAs in total and polysomal fractions.** Temporal real-time RT-PCR profile of selected 5′-TOP mRNA expression in the total RNA (black line) and polysomal RNA (red line) fractions from mouse liver. For each time point, data are mean ± standard error of the mean (SEM) obtained from four independent animals. In addition to three ribosomal protein mRNA, which are known to have a 5′-TOP and be regulated by TORC1 [19], we selected also *Receptor of ACtivated protein Kinase C 1* (*Rack1*) or *Guanine Nucleotide Binding protein (G protein), Beta polypeptide 2-Like 1* (*Gnb2l1*), a ribosome constituent [80] known to be regulated by TORC1 [81], which also plays a role in circadian clock regulation [82]. However, a potential role of *Rack1* rhythmic translation on the circadian clock is not documented. The zeitgeber times (ZT) at which the animals were sacrificed are indicated on each panel.
(TIF)

Figure S11 **Temporal expression of ribosomal proteins in mouse liver.** Mean ± standard error of the mean (SEM) (*n* = 3) densitometric values of the Western blot data depicted in Figure 3D were represented according to the zeitgeber time. Statistical analysis of these data is given in Table S2.
(TIF)

Figure S12 **Temporal expression of UBF1 in WT, and in** ***Cry1/Cry2*** **KO, and** ***Bmal1*** **KO mouse liver.** (A) Mean ± standard error of the mean (SEM) (*n* = 3) densitometric values of the Western blot data depicted in Figure 4B were represented according to the zeitgeber time. Statistical analysis of these data is given in Table S2. (B) Mean ± SEM (*n* = 2) densitometric values of the Western blot data depicted in Figure 4C (*Cry1*/*Cry2* KO mice) and 4D (*Bmal1* KO mice) were represented according to the zeitgeber time. Statistical analysis of these data is given in Tables S7 and S8, respectively.
(TIF)

Figure S13 **Activation of the TORC1, PI3K, and ERK pathways in** ***Cry1/Cry2*** **and** ***Bmal1*** **KO mice kept in constant darkness.** (A) Temporal phosphorylation of RPS6, AKT, and ERK in mouse mutant liver. *Cry1*/*Cry2* and *Bmal1* KO mice were placed in constant darkness for 3 d and then sacrificed every 4 h during a 24-h period. Total liver extracts were used for Western blotting. The circadian (CT) times at which the animals were sacrificed are indicated on the top of the figures. As expected, rhythmic activation of the three pathways is lost under these conditions. (B) Six *Cry1*/*Cry2* KO mice were kept in constant darkness for one week and then sacrificed at CT12. Phosphorylation of RPS6, AKT and ERK were evaluated by Western blotting on total liver extracts. We observed as expected in these conditions a high degree of variability in the activation of the three pathways, probably due to the arrhythmic food consumption of the animals. However, the ERK pathway seems to be less affected. A quantification of these data is given on the right part of the figure. Naphtol blue black staining of the membranes was used as a loading control.
(TIF)

Figure S14 **Diurnal expression of genes encoding proteins involved in TORC1 complex, mRNA translation initiation and RPs synthesis in WT and** ***Cry1*** **/** ***Cry2*** **KO mice.** Temporal real-time RT-PCR expression of genes encoding proteins involved in TORC1 complex (*mTor* and *Raptor*), mRNA translation initiation (*Eif4b* and *Eif4ebp3*), and RP synthesis (*Rpl32* and *Rpl34* pre-mRNA) in total RNA from WT (black line) and *Cry1*/*Cry2* KO (red line) mouse liver. For each time point, data are mean ± standard error of the mean (SEM) obtained from four (WT) and three (KO) independent animals. The zeitgeber times (ZT) at which the animals were sacrificed are indicated on each panel.
(TIF)

Figure S15 **Diurnal expression of genes encoding proteins involved in TORC1 complex, mRNA translation initiation, and RP synthesis in WT and** ***Bmal1*** **KO mice.** Temporal real-time RT-PCR expression of genes encoding proteins involved in TORC1 complex (*mTor* and *Raptor*), mRNA translation initiation (*Eif4b* and *Eif4ebp3*), and RP synthesis (*Rpl32* and *Rpl34* pre-mRNA) in total RNA from WT (black line) and *Bmal1* KO (red line) mouse liver. For each time point, data are mean ± standard error of the mean (SEM) obtained from two independent animals. The zeitgeber times (ZT) at which the animals were sacrificed are indicated on each panel.
(TIF)

Figure S16 **Temporal expression and phosphorylation of proteins involved in translational initiation, signaling pathways activation, and ribosome biogenesis in** ***Cry1*** **/** ***Cry2*** **KO mice.** (A) Mean ± standard error of the mean (SEM) (*n* = 2) densitometric values of the Western blot data depicted in Figure 6A were represented according to the zeitgeber time. (B) Mean ± SEM (*n* = 2) densitometric values of the Western blot data depicted in Figure 6B were represented according to the zeitgeber time. Statistical analysis of these data is given in Table S7. It is interesting to note that expression of EIF4E is slightly increased in the KO (Student's *t*-test *p*≤0.05), in agreement with the increased mRNA expression. It is also the case for RPS6 whose expression increase like most of the other RP proteins (Student's *t*-test *p*≤3×10^−6^).
(TIF)

Figure S17 **Temporal expression and phosphorylation of proteins involved in translational initiation, signaling pathways activation, and ribosome biogenesis in** ***Bmal1*** **KO mice.** (A) Mean ± standard error of the mean (SEM) (*n* = 2) densitometric values of the Western blot data depicted in Figure 6C were represented according to the zeitgeber time. (B) Mean ± SEM (*n* = 2) densitometric values of the Western blot data depicted in Figure 6D were represented according to the zeitgeber time. Statistical analysis of these data is given in Table S8.
(TIF)

Figure S18 **Example of polysomes purification profile.** Optic density at 260 nm of the 45 sub-fractions obtained after ultracentrifugation of liver extract from mouse sacrificed at ZT8. These fractions are then pooled in ten fractions and the fractions 1 and 2 are pooled to obtain the polysomal fraction used in microarray and RT-PCR experiments.
(TIF)

Table S1 **Cosinor statistical values related to rhythmic mRNA expression of genes coding for proteins involved in mRNA translation, TORC1 complex, and ribosome biogenesis.** A Cosinor statistical analysis was applied to the rhythmic datasets corresponding to the respective expression of the indicated mRNA measured by quantitative PCR in WT mice and shown on Figures 1, 4, and S3.
(DOC)

Table S2 **Cosinor statistical values related to rhythmic expression and phosphorylations of proteins involved in mRNA translation, TORC1 complex, and ribosome biogenesis.** A Cosinor statistical analysis was applied to the rhythmic datasets corresponding to the respective expression of the indicated proteins measured by Western blots quantification in WT mice and shown on Figures S1, S2, S11, and S12.
(DOC)

Table S3 **Affymetrix microarray probes presenting a rhythmic polysomal/total RNA ratio and in phase with TORC1 activation (complement to** **Figure 3A** **).** Affymetrix microarray probes presenting a rhythmic polysomal/total RNA ratio and in phase with TORC1 activation were classified according to the phase of the maximum value (all include between ZT14 and ZT18).
(XLS)

Table S4 **Functions of the genes presenting a rhythmic total/polysomal RNA ratio.** Most of the genes found regulated at the translational level are known 5′-TOP containing genes. They include almost all the RP coding genes: 28 of the 32 small RP genes and 42 of the 47 large RP genes expressed in mouse [83] are found on the list. The list also includes known 5′-TOP mRNA encoding proteins involved in the regulation of translation: translation initiation factors of the class 2, 3, and 4, first class of translation elongation factors, and poly-A binding proteins [19]. In addition, the list contains genes encoding proteins involved at different steps of translational regulation and ribosome biogenesis: NPM1, a chaperone protein involved in ribosome assembly and rRNA maturation [84]; CCT4, a member of the chaperonin complex that plays a role in ribosome biogenesis [85]; TPT1, a guanine nucleotide exchanger that controls TORC1 activity through regulation of the RHEB GTPase [86]; IGBP1, a regulatory subunit of protein phosphatase 2A that modulates TORC1 activity [87]; PFDN5, a chaperone protein that modulates MYC activity [88]; a transcription factor involved in rRNA and RP mRNA transcription [89]; AHCY, a *S*-adenosyl homocysteine hydrolase that regulates translation also through modulation of MYC activity [90]; GNB2L1 or RACK1, a scaffold protein that interacts with and modulates ribosome activity [80]; UBA52, a protein constitutes by the fusion of a ribosomal protein and ubiquitin [91]; The remaining genes encode proteins with unknown function in translation regulation.
(DOC)

Table S5 **Cosinor statistical values related to rhythmic mRNA expression of genes coding for proteins involved in mRNA translation, TORC1 complex, and ribosome biogenesis in WT and** ***Cry1*** **/** ***Cry2*** **KO mice.** A Cosinor statistical analysis was applied to the rhythmic datasets corresponding to the respective expression of the indicated mRNA measured by quantitative PCR in WT and *Cry1*/*Cry2* KO mice and shown on Figures 4, 5, and S14.
(DOC)

Table S6 **Cosinor statistical values related to rhythmic mRNA expression of genes coding for proteins involved in mRNA translation, TORC1 complex and ribosome biogenesis in WT and** ***Bmal1*** **KO mice.** A Cosinor statistical analysis was applied to the rhythmic datasets corresponding to the respective expression of the indicated mRNA measured by quantitative PCR in WT and *Bmal1* KO mice and shown on Figures 4, 5, and S15.
(DOC)

Table S7 **Cosinor statistical values related to rhythmic expression and phosphorylation of proteins involved in mRNA translation, TORC1 complex and ribosome biogenesis in WT and** ***Cry1*** **/** ***Cry2*** **KO mice.** A Cosinor statistical analysis was applied to the rhythmic datasets corresponding to the respective expression of the indicated proteins measured by Western blots quantification in WT and *Cry1*/*Cry2* KO mice and shown on Figures S12 and S16.
(DOC)

Table S8 **Cosinor statistical values related to rhythmic expression and phosphorylation of proteins involved in mRNA translation, TORC1 complex, and ribosome biogenesis in WT and** ***Bmal1*** **KO mice.** A Cosinor statistical analysis was applied to the rhythmic datasets corresponding to the respective expression of the indicated proteins measured by Western blots quantification in WT and *Bmal1* KO mice and shown on Figures S12 and S17.
(DOC)

Table S9 **Taqman probes used for real-time PCR (Applied Biosystems).**
(DOC)

Table S10 **Sequences of the primers used for SYBR Green real-time PCR.**
(DOC)

Table S11 **References of the antibodies used for Western blotting** [**92**],[**93**] **.**
(DOC)

## Abbreviations

AMPK: adenosine monophosphate-activated protein kinase
ERK: extracellular signal-regulated protein kinase
KO: knockout
PI3K: phosphoinositide 3-kinase
RP: ribosomal protein
RPS6: ribosomal protein S6
RT: reverse transcription
SCN: suprachiasmatic nucleus
TOP: terminal oligopyrimidine tract
TORC1: target of rapamycin complex 1
TSC: tuberous sclerosis protein complex
UBF: upstream binding factor
WT: wild type
