# Supplementary figures and images for: The Circadian Clock Coordinates Ribosome Biogenesis
Source: PLoS Biol. 2013 Jan 3;11(1):e1001455. doi: 10.1371/journal.pbio.1001455 (PMC3536797; doi:10.1371/journal.pbio.1001455)

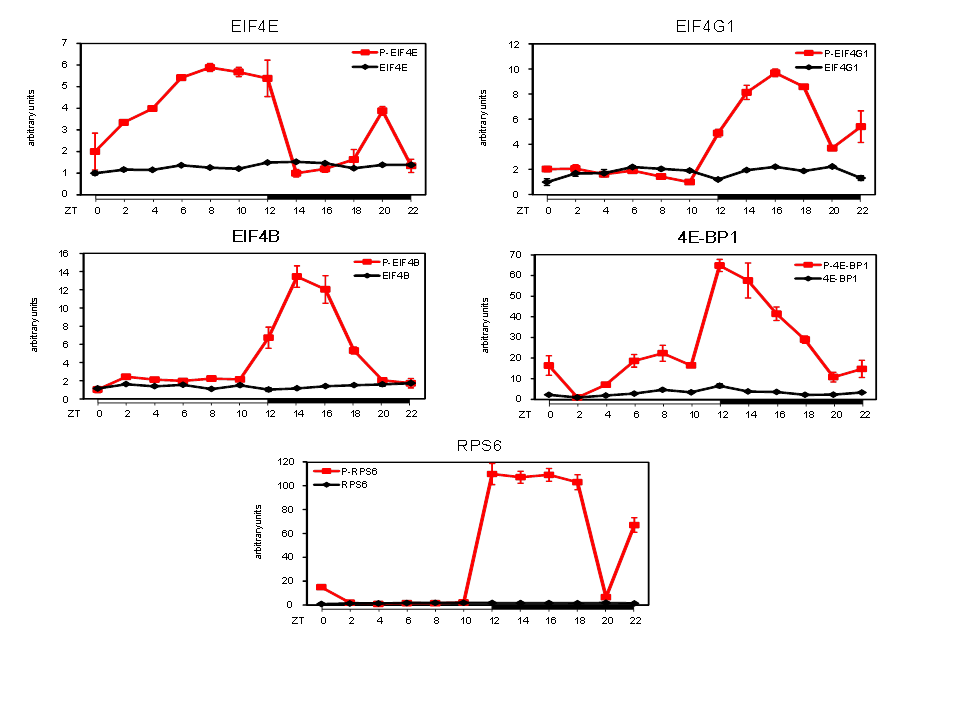

Supplement: Figure S1 — Temporal expression and phosphorylation of translation initiation factors in WT mice. Mean ± standard error of the mean (SEM) (n = 3) densitometric values of the Western blot data depicted in Figure 1B were represented according to the zeitgeber time. Statistical analysis of these data is given in Table S2. (TIF) [file pbio.1001455.s001.tif]

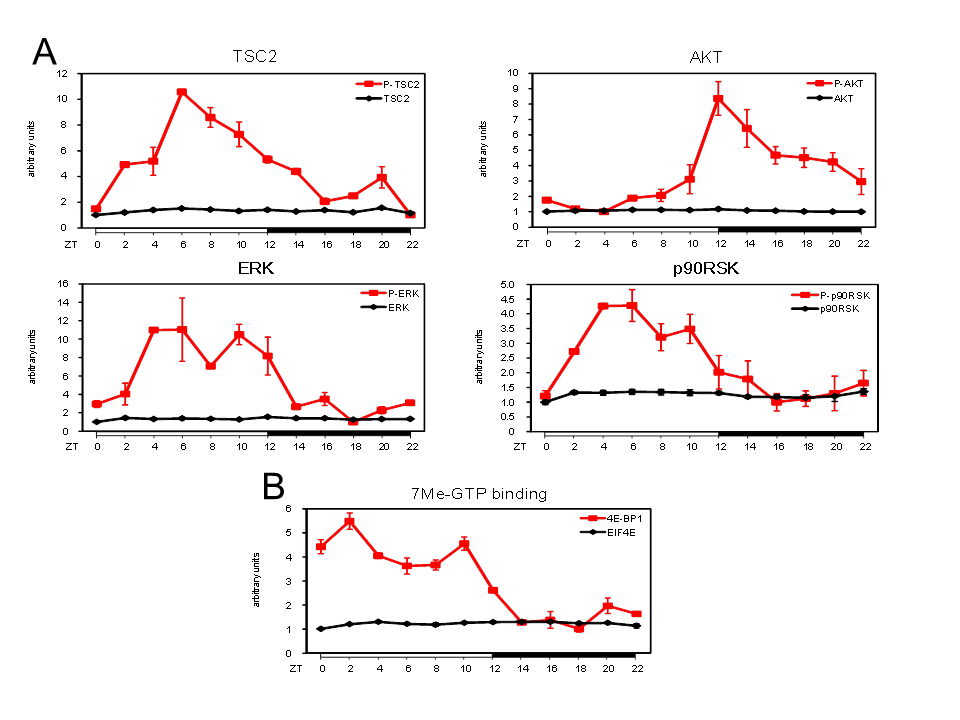

Supplement: Figure S2 — Temporal expression and phosphorylation of proteins involved in signaling pathways activation and translational initiation in WT mice. (A) Mean ± standard error of the mean (SEM) (n = 3) densitometric values of the Western blot data depicted in Figure 2A were represented according to the zeitgeber time. (B) Mean ± SEM (n = 2) densitometric values of the Western blot data depicted in Figure 2B were represented according to the zeitgeber time. Statistical analysis of these data is given in Table S2. (TIF) [file pbio.1001455.s002.tif]

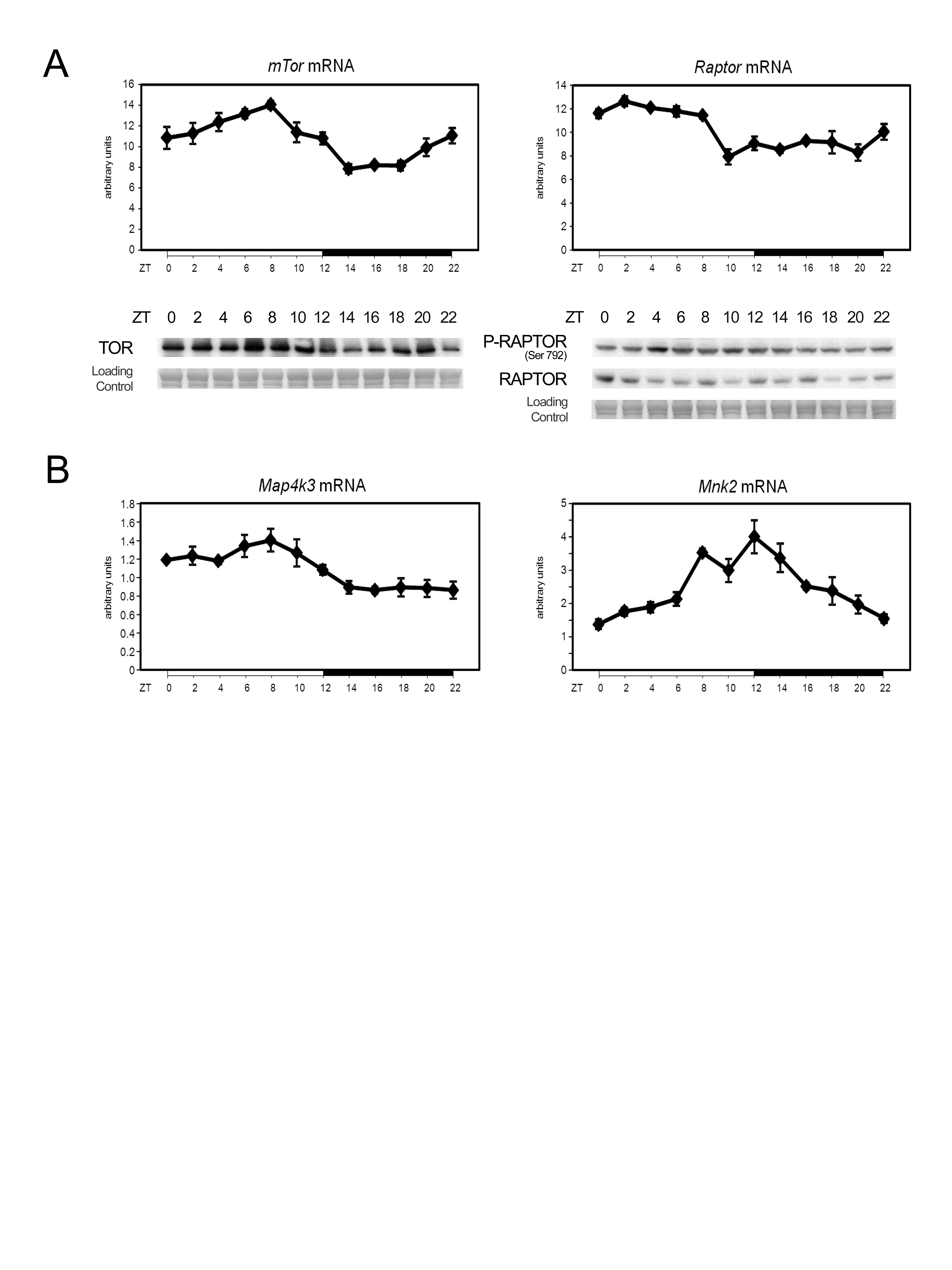

Supplement: Figure S3 — Temporal expression of TORC1 components and of kinases regulating TORC1 and EIF4E activities in WT mice. (A) Temporal expression of the TORC1 components mTor and Raptor at the mRNA level (upper panel) and protein level (lower panel) in mouse liver. mRNA expressions were measured by real-time RT-PCR. For each time point, data are mean ± standard error of the mean (SEM) obtained from four independent animals. Expression of mTOR and RAPTOR and its phosphorylation on Serine 792 were measured by Western blot on total extracts. The phosphorylation of RAPTOR on Serine 792 by AMPK has been shown to reduce TORC1 activity [75] and contributes to the inhibition of TORC1 during the day. Naphtol blue black staining of the membranes was used as a loading control. (B) Temporal expression of Map4k3 (left panel) and Mnk2 mRNA (right panel) in mouse liver. mRNA expressions were measured by real-time RT-PCR. For each time point, data are mean ± SEM obtained from four independent animals. MAP4K3 plays a role in the activation of TORC1 by amino acids [76], whereas MNK2 is involved in the ERK signaling cascade leading to the phosphorylation of EIF4E, which can play a role in 5′-TOP mRNA translation [9]. (TIF) [file pbio.1001455.s003.tif]

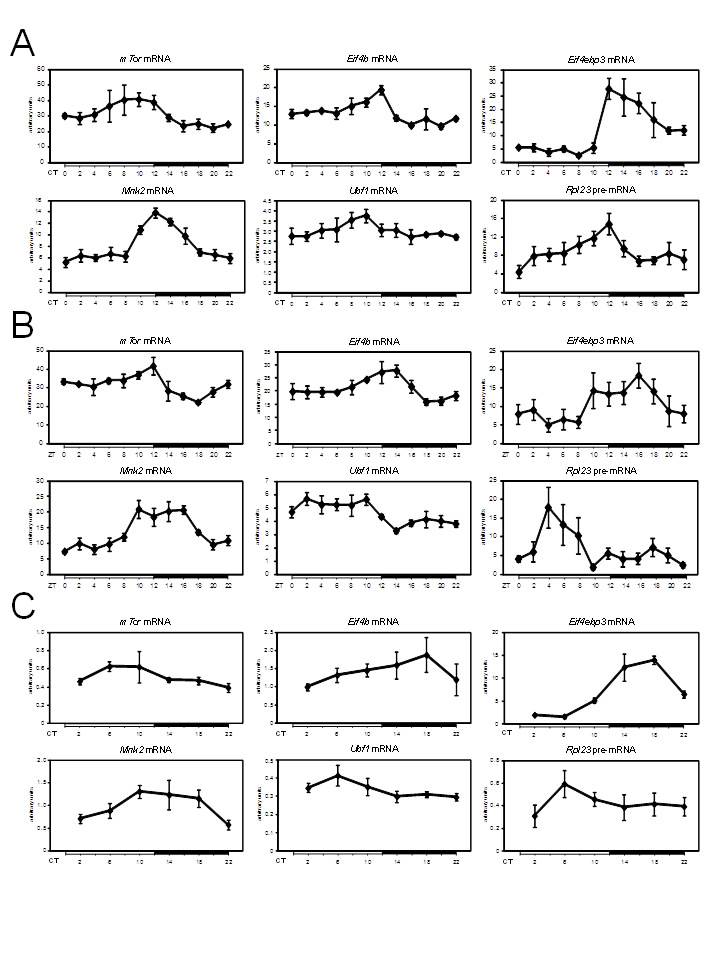

Supplement: Figure S4 — Rhythmic expression of mRNA encoding translation initiation factors ( Eif4b , Eif4ebp3 ), the TORC1 complex component mTor , the kinase activating these factors Mnk2 , and proteins involved in rRNA synthesis ( Ubf1 ) and ribosome biogenesis ( Rpl23 ) is independent of food and light. (A) Temporal expression in constant darkness. (B) Temporal expression during starvation. (C) Temporal expression during starvation in constant darkness. mRNA expressions were measured by real-time RT-PCR. For each time point, data are mean ± SEM obtained from three independent animals. The circadian (CT) or zeitgeber (ZT) times at which the animals were sacrificed are indicated on the bottom of the figures. (TIF) [file pbio.1001455.s004.tif]

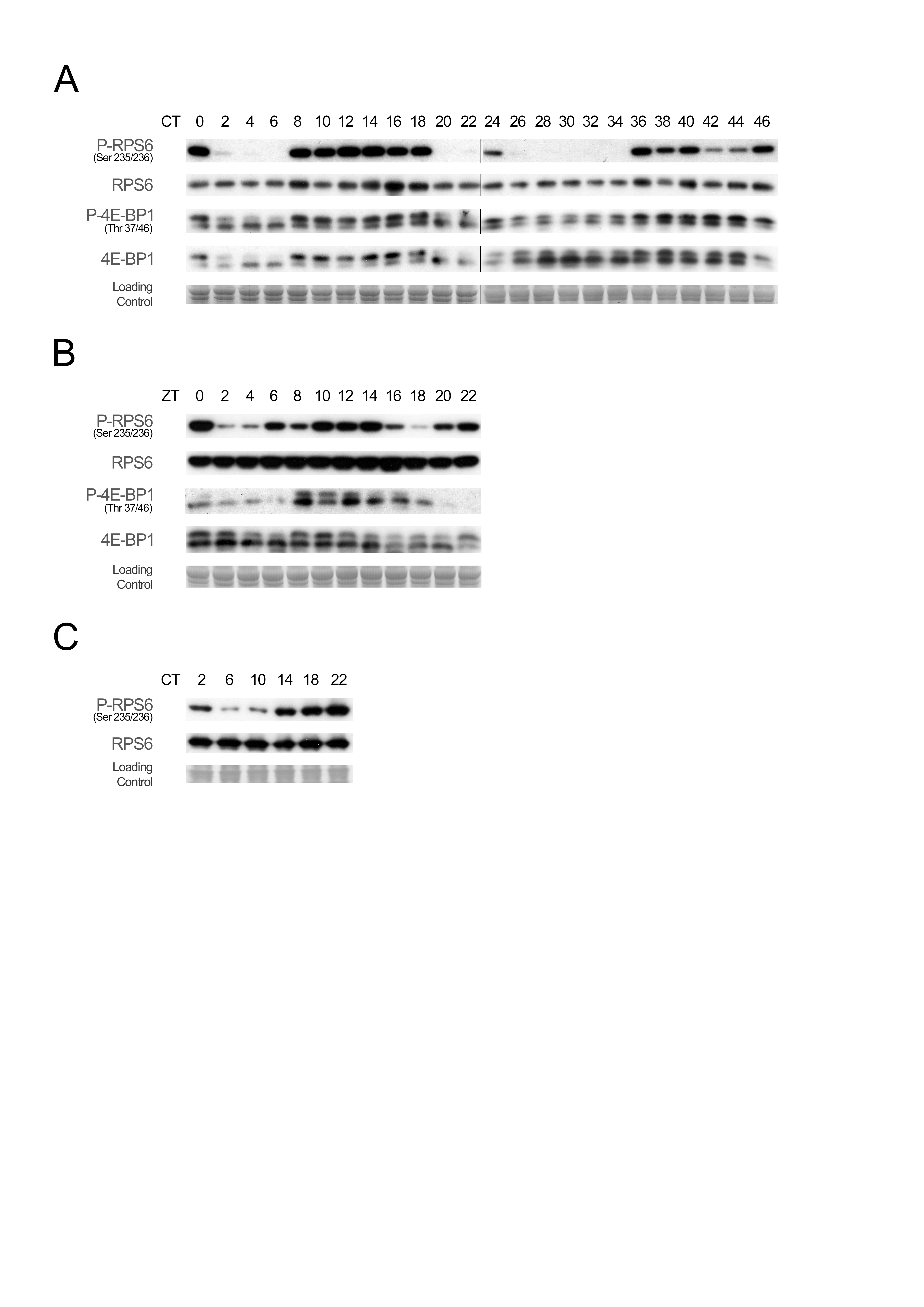

Supplement: Figure S5 — Rhythmic activation of TORC1 still occurs in constant conditions. (A) Temporal phosphorylation of TORC1 substrates during 48 h in constant darkness. The lines through gels indicate where the images have been cropped. (B) Temporal phosphorylation of TORC1 substrates during starvation. As reported [14], the period of activation seems to be shorter in these conditions. Interestingly, this activation is antiphasic with the rhythmic activation of autophagy in mouse liver [15], a process inhibited by TORC1 but able to generate amino acids that can in turn activate TORC1 [16]. (C) Temporal phosphorylation of the TORC1 substrate RPS6 during starvation in constant darkness. Temporal expression and phosphorylation of RPS6 and 4E-BP1 were measured by Western blot on total extracts. Naphtol blue black staining of the membranes was used as a loading control.The circadian (CT) or zeitgeber (ZT) times at which the animals were sacrificed are indicated on the top of the figures. (TIF) [file pbio.1001455.s005.tif]

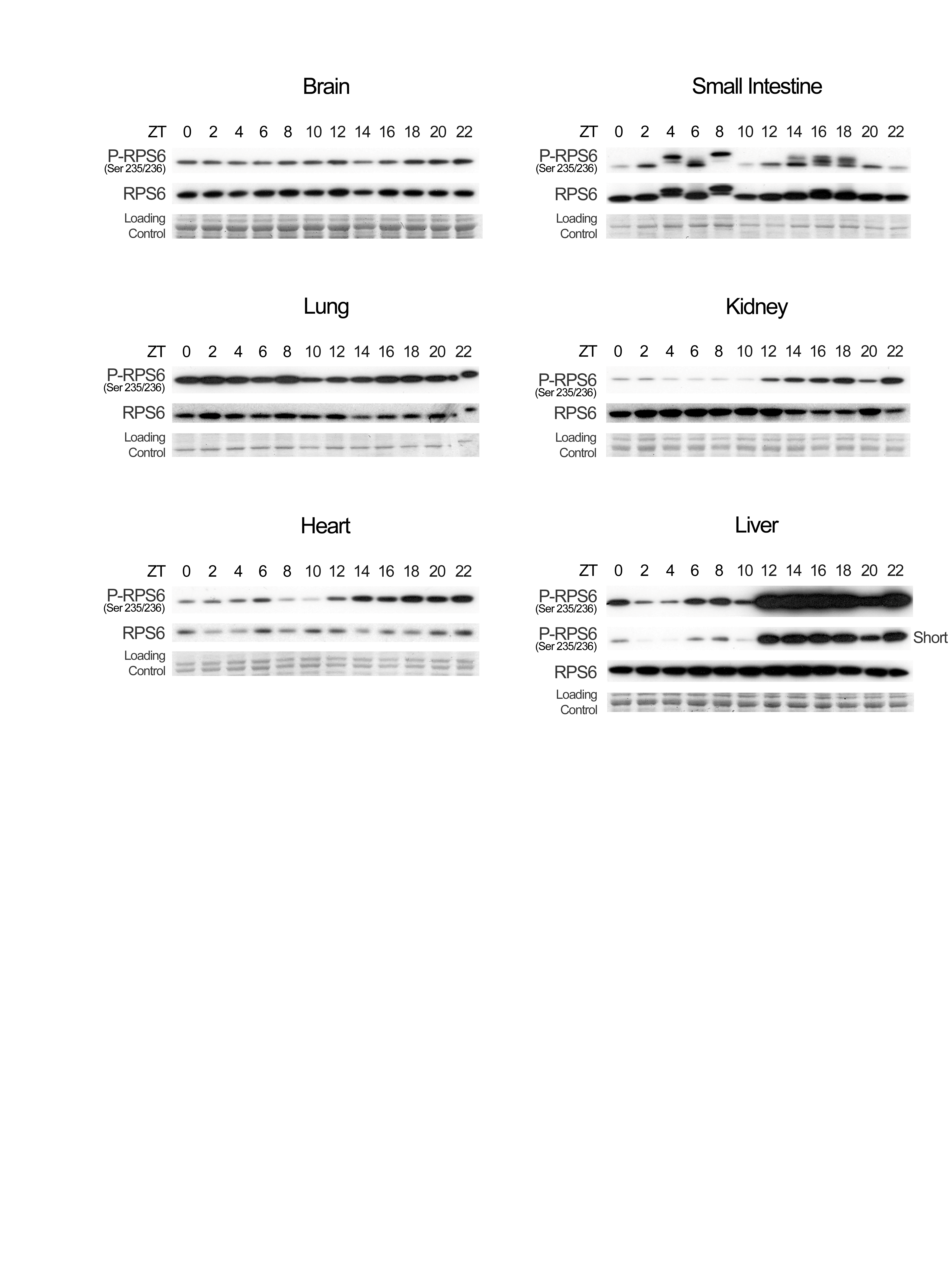

Supplement: Figure S6 — Rhythmic activation of TORC1 in different mouse organs. Temporal activation of the TORC1 pathway in mouse organs, revealed by phosphorylation of RPS6. As in the liver, this rhythmic activation is kept in kidney and heart, nevertheless with reduced amplitude (indicated by the blot with a shortest exposure). However, TORC1 activation is constant in brain, lung, and small intestine, suggesting that nutriment availability due to rhythmic feeding is not sufficient to explain this phenomenon. The zeitgeber times (ZT) at which the animals were sacrificed are indicated on each panel. Naphtol blue black staining of the membranes was used as a loading control. (TIF) [file pbio.1001455.s006.tif]

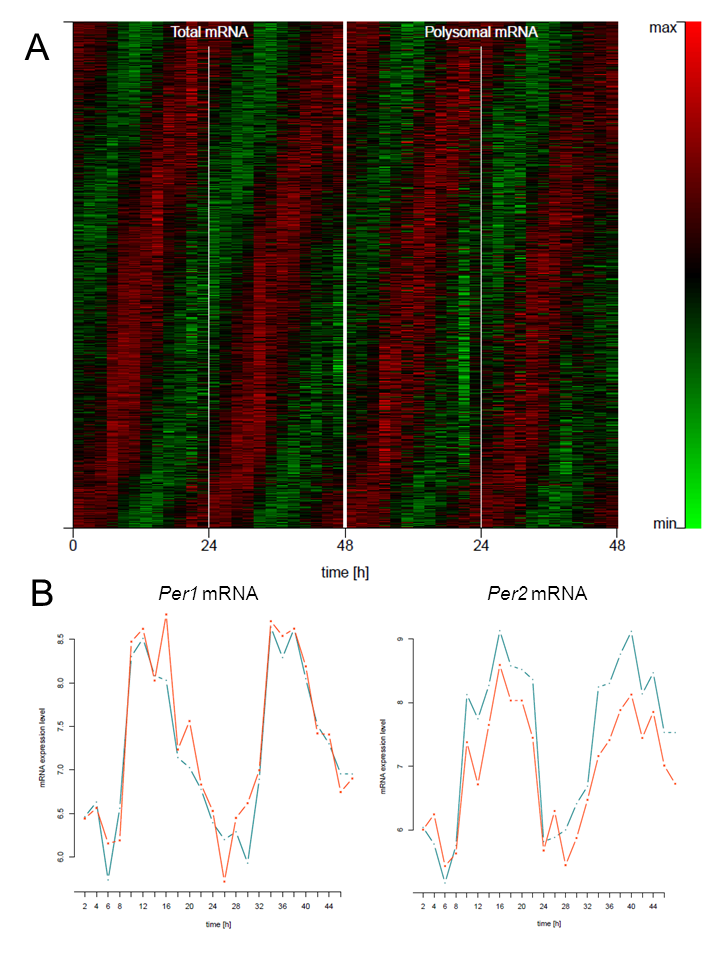

Supplement: Figure S8 — The temporal profiles of polysomal mRNAs closely follow that of total mRNAs for most circadian genes, as exemplified by the Period genes. (A) Temporal profiles ordered by phase in total (left panel) and polysomal RNA (right panel) fractions of microarray probes presenting a rhythmic profile in total mRNA fraction. Data were mean centered and standardized. Log-ratios are color-coded so that red indicates high and green low relative levels of mRNA. For most of the probes, the profiles are strikingly similar in the two fractions, indicating constant translational efficacy along the day. (B) Temporal expression of Per1 (left panel) and Per2 (right panel) mRNAs in polysomal (red line) and total (blue line) RNA fractions. Data are represented in log scale without any additional normalization than the one provided by the Affymetrix software. Although a regulation of PER1 expression at the translational level has been proposed [78],[79], this hypothesis is not confirmed by our in vivo data as the two profiles are extremely similar. (TIF) [file pbio.1001455.s008.tif]

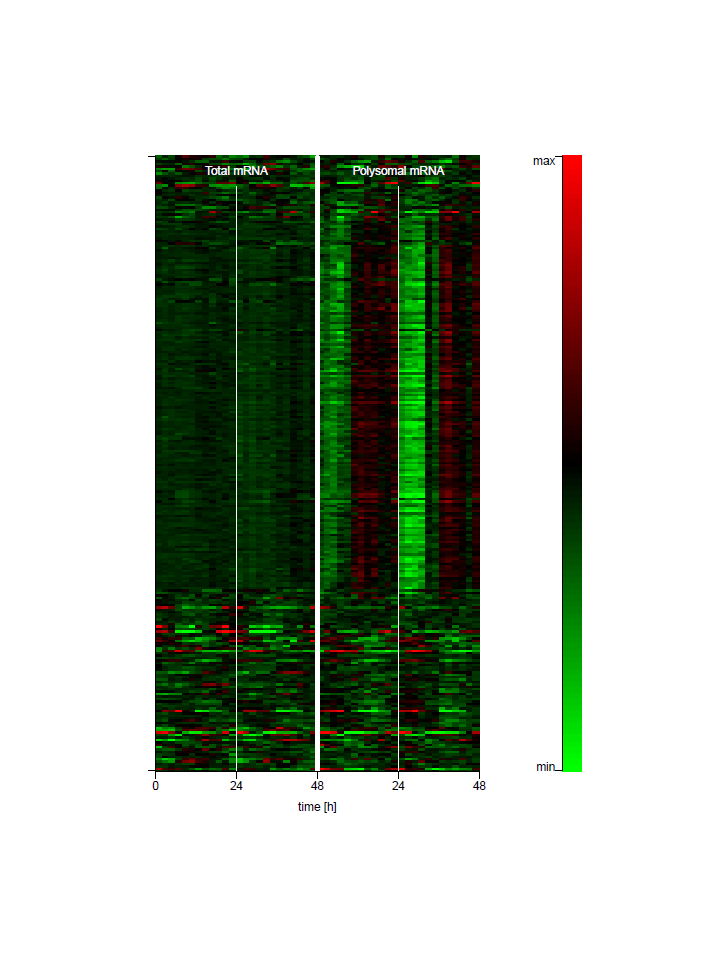

Supplement: Figure S9 — Comparative diurnal expression profile of RNA in total and polysomal fractions. Temporal profiles of total RNA (left panel) and polysomal RNA (right panel) fractions of microarray probes presenting a rhythmic polysomal/total RNA ratio. The profiles are ordered by the phase of the polysomal/total ratio phase. Data were mean centered and standardized. Log-ratios are color-coded so that red indicates high and green low relative levels of mRNA. (TIF) [file pbio.1001455.s009.tif]

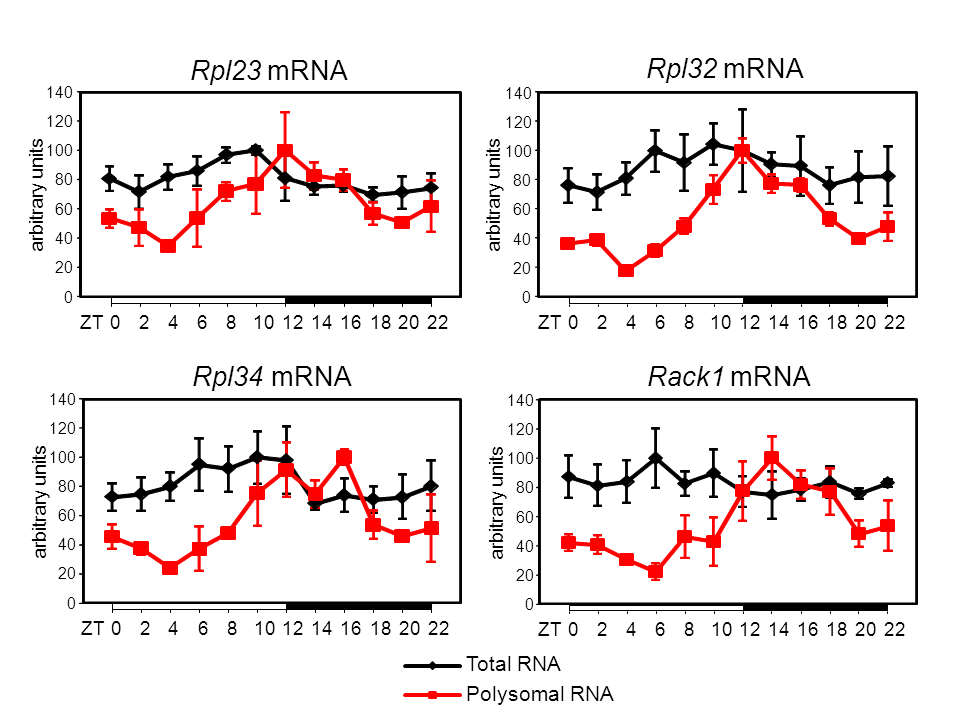

Supplement: Figure S10 — Diurnal expression of selected 5′-TOP mRNAs in total and polysomal fractions. Temporal real-time RT-PCR profile of selected 5′-TOP mRNA expression in the total RNA (black line) and polysomal RNA (red line) fractions from mouse liver. For each time point, data are mean ± standard error of the mean (SEM) obtained from four independent animals. In addition to three ribosomal protein mRNA, which are known to have a 5′-TOP and be regulated by TORC1 [19], we selected also Receptor of ACtivated protein Kinase C 1 (Rack1) or Guanine Nucleotide Binding protein (G protein), Beta polypeptide 2-Like 1 (Gnb2l1), a ribosome constituent [80] known to be regulated by TORC1 [81], which also plays a role in circadian clock regulation [82]. However, a potential role of Rack1 rhythmic translation on the circadian clock is not documented. The zeitgeber times (ZT) at which the animals were sacrificed are indicated on each panel. (TIF) [file pbio.1001455.s010.tif]

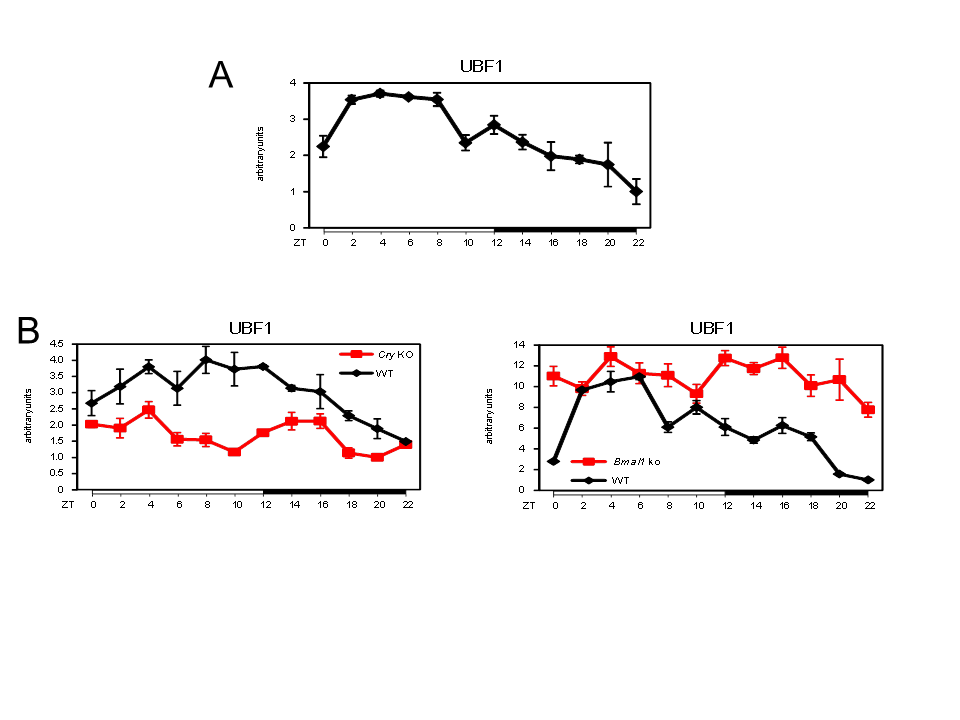

Supplement: Figure S12 — Temporal expression of UBF1 in WT, and in Cry1/Cry2 KO, and Bmal1 KO mouse liver. (A) Mean ± standard error of the mean (SEM) (n = 3) densitometric values of the Western blot data depicted in Figure 4B were represented according to the zeitgeber time. Statistical analysis of these data is given in Table S2. (B) Mean ± SEM (n = 2) densitometric values of the Western blot data depicted in Figure 4C (Cry1/Cry2 KO mice) and 4D (Bmal1 KO mice) were represented according to the zeitgeber time. Statistical analysis of these data is given in Tables S7 and S8, respectively. (TIF) [file pbio.1001455.s012.tif]

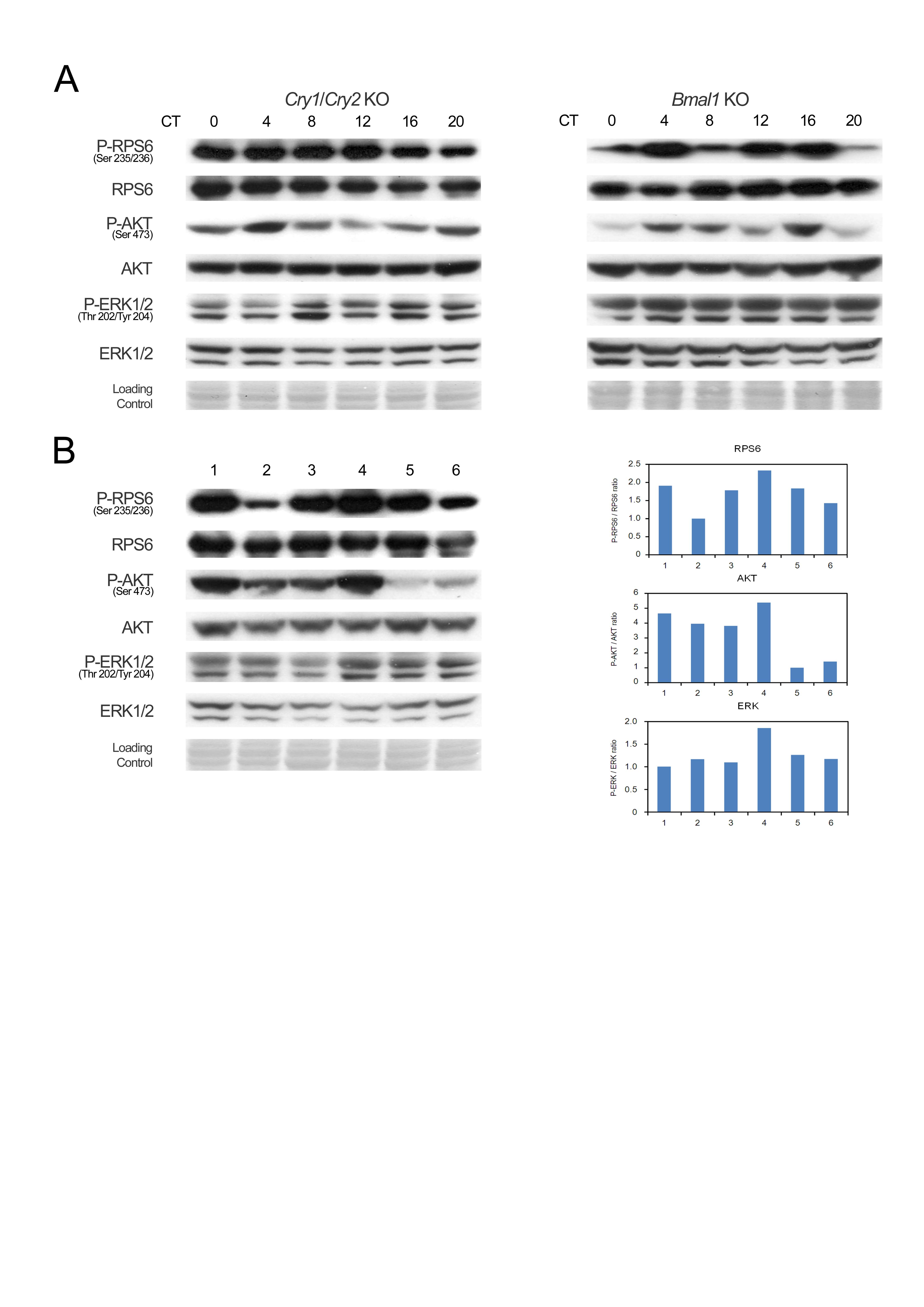

Supplement: Figure S13 — Activation of the TORC1, PI3K, and ERK pathways in Cry1/Cry2 and Bmal1 KO mice kept in constant darkness. (A) Temporal phosphorylation of RPS6, AKT, and ERK in mouse mutant liver. Cry1/Cry2 and Bmal1 KO mice were placed in constant darkness for 3 d and then sacrificed every 4 h during a 24-h period. Total liver extracts were used for Western blotting. The circadian (CT) times at which the animals were sacrificed are indicated on the top of the figures. As expected, rhythmic activation of the three pathways is lost under these conditions. (B) Six Cry1/Cry2 KO mice were kept in constant darkness for one week and then sacrificed at CT12. Phosphorylation of RPS6, AKT and ERK were evaluated by Western blotting on total liver extracts. We observed as expected in these conditions a high degree of variability in the activation of the three pathways, probably due to the arrhythmic food consumption of the animals. However, the ERK pathway seems to be less affected. A quantification of these data is given on the right part of the figure. Naphtol blue black staining of the membranes was used as a loading control. (TIF) [file pbio.1001455.s013.tif]

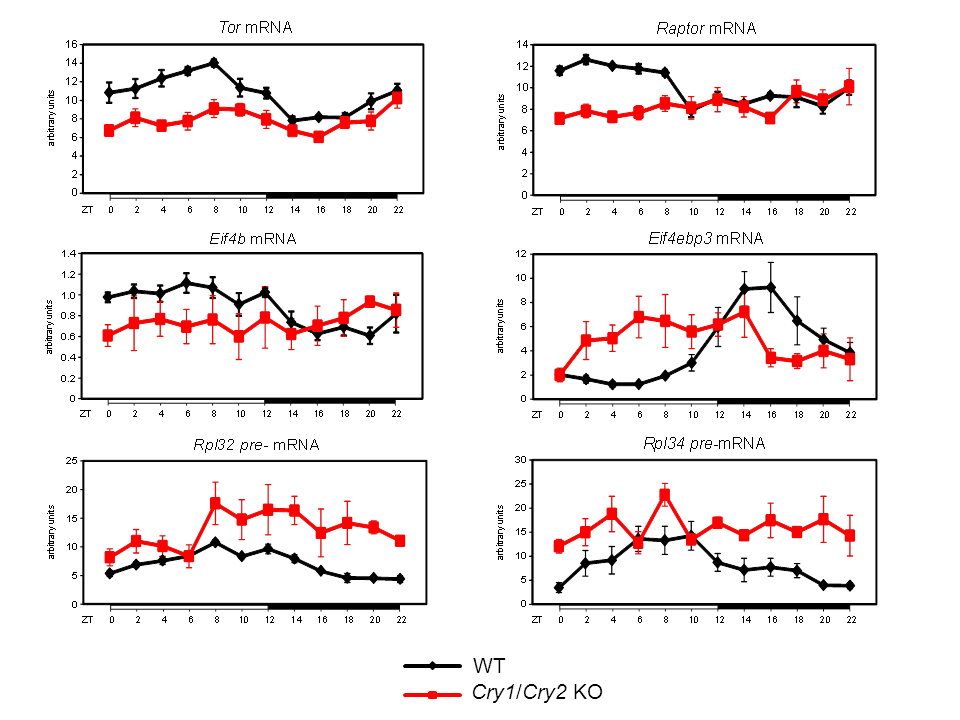

Supplement: Figure S14 — Diurnal expression of genes encoding proteins involved in TORC1 complex, mRNA translation initiation and RPs synthesis in WT and Cry1 / Cry2 KO mice. Temporal real-time RT-PCR expression of genes encoding proteins involved in TORC1 complex (mTor and Raptor), mRNA translation initiation (Eif4b and Eif4ebp3), and RP synthesis (Rpl32 and Rpl34 pre-mRNA) in total RNA from WT (black line) and Cry1/Cry2 KO (red line) mouse liver. For each time point, data are mean ± standard error of the mean (SEM) obtained from four (WT) and three (KO) independent animals. The zeitgeber times (ZT) at which the animals were sacrificed are indicated on each panel. (TIF) [file pbio.1001455.s014.tif]

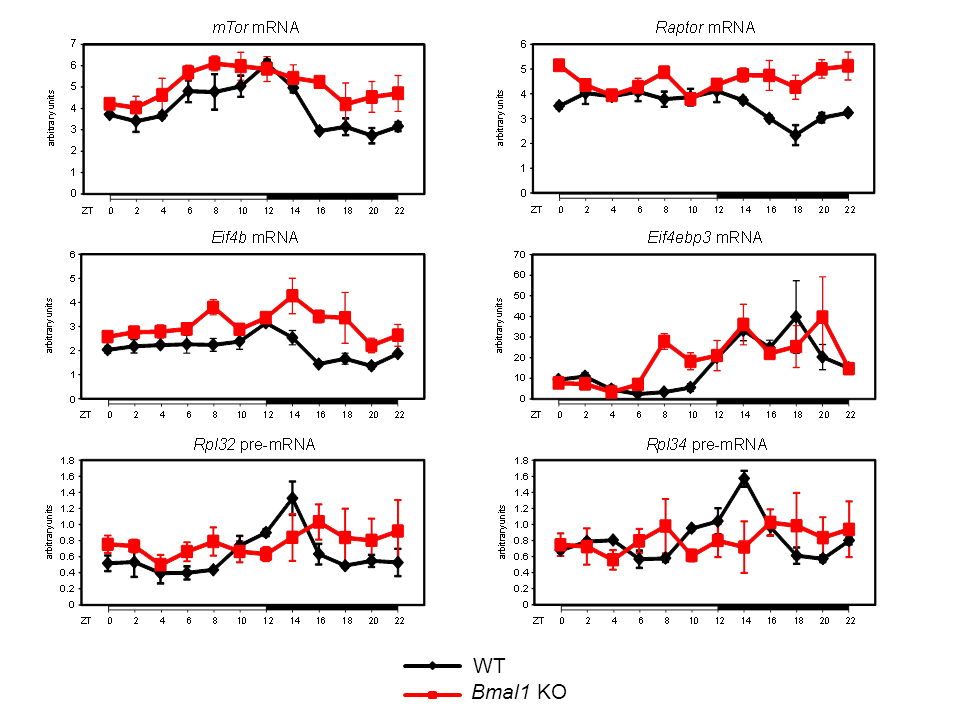

Supplement: Figure S15 — Diurnal expression of genes encoding proteins involved in TORC1 complex, mRNA translation initiation, and RP synthesis in WT and Bmal1 KO mice. Temporal real-time RT-PCR expression of genes encoding proteins involved in TORC1 complex (mTor and Raptor), mRNA translation initiation (Eif4b and Eif4ebp3), and RP synthesis (Rpl32 and Rpl34 pre-mRNA) in total RNA from WT (black line) and Bmal1 KO (red line) mouse liver. For each time point, data are mean ± standard error of the mean (SEM) obtained from two independent animals. The zeitgeber times (ZT) at which the animals were sacrificed are indicated on each panel. (TIF) [file pbio.1001455.s015.tif]

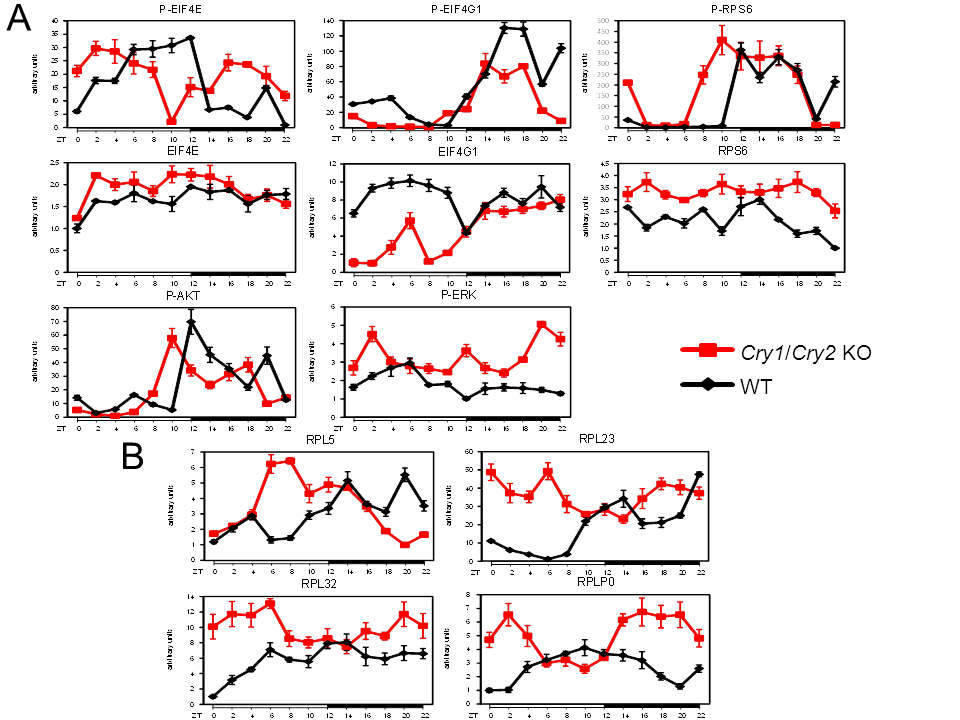

Supplement: Figure S16 — Temporal expression and phosphorylation of proteins involved in translational initiation, signaling pathways activation, and ribosome biogenesis in Cry1 / Cry2 KO mice. (A) Mean ± standard error of the mean (SEM) (n = 2) densitometric values of the Western blot data depicted in Figure 6A were represented according to the zeitgeber time. (B) Mean ± SEM (n = 2) densitometric values of the Western blot data depicted in Figure 6B were represented according to the zeitgeber time. Statistical analysis of these data is given in Table S7. It is interesting to note that expression of EIF4E is slightly increased in the KO (Student's t-test p≤0.05), in agreement with the increased mRNA expression. It is also the case for RPS6 whose expression increase like most of the other RP proteins (Student's t-test p≤3×10^−6^). (TIF) [file pbio.1001455.s016.tif]

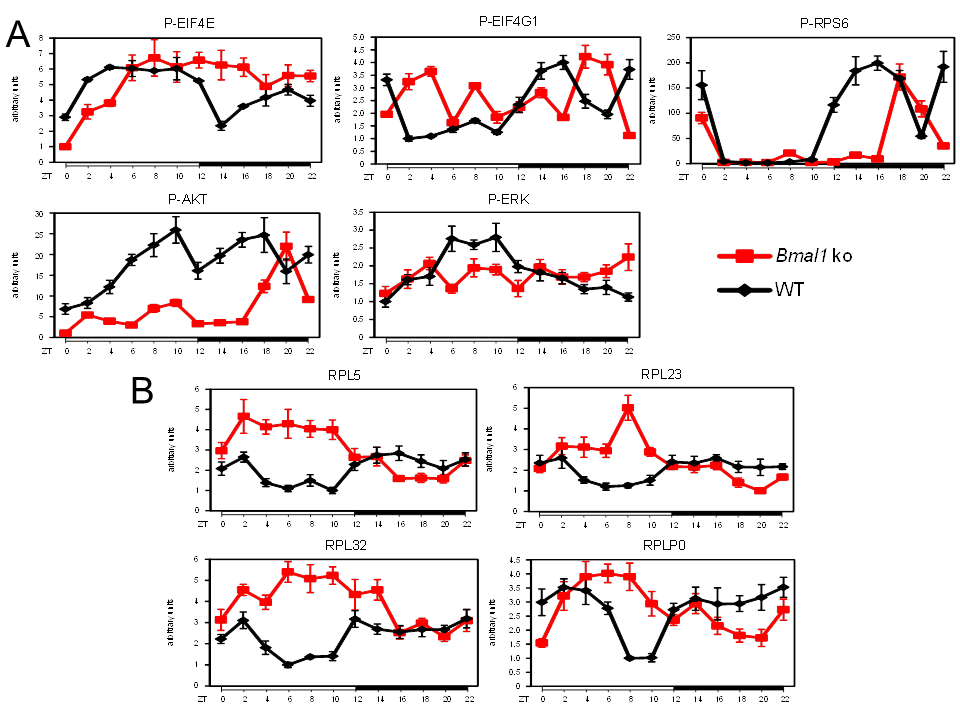

Supplement: Figure S17 — Temporal expression and phosphorylation of proteins involved in translational initiation, signaling pathways activation, and ribosome biogenesis in Bmal1 KO mice. (A) Mean ± standard error of the mean (SEM) (n = 2) densitometric values of the Western blot data depicted in Figure 6C were represented according to the zeitgeber time. (B) Mean ± SEM (n = 2) densitometric values of the Western blot data depicted in Figure 6D were represented according to the zeitgeber time. Statistical analysis of these data is given in Table S8. (TIF) [file pbio.1001455.s017.tif]

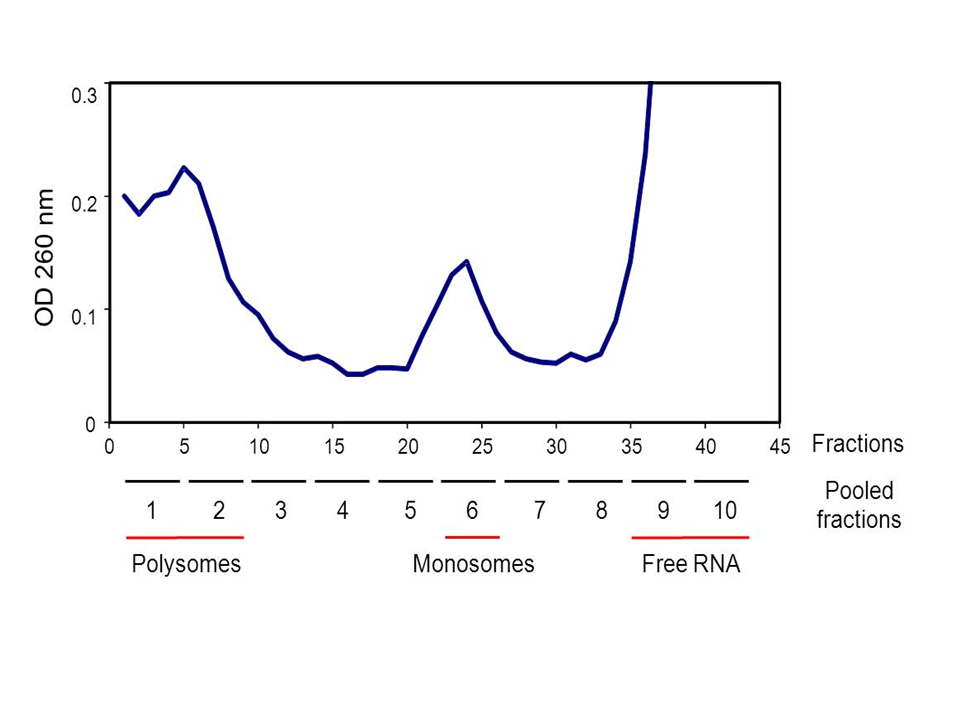

Supplement: Figure S18 — Example of polysomes purification profile. Optic density at 260 nm of the 45 sub-fractions obtained after ultracentrifugation of liver extract from mouse sacrificed at ZT8. These fractions are then pooled in ten fractions and the fractions 1 and 2 are pooled to obtain the polysomal fraction used in microarray and RT-PCR experiments. (TIF) [file pbio.1001455.s018.tif]
