# Supplementary material for: The Circadian Clock Coordinates Ribosome Biogenesis
Source: PLoS Biol. 2013 Jan 3;11(1):e1001455. doi: 10.1371/journal.pbio.1001455 (PMC3536797; doi:10.1371/journal.pbio.1001455)
Supplement: Table S1 — Cosinor statistical values related to rhythmic mRNA expression of genes coding for proteins involved in mRNA translation, TORC1 complex, and ribosome biogenesis. A Cosinor statistical analysis was applied to the rhythmic datasets corresponding to the respective expression of the indicated mRNA measured by quantitative PCR in WT mice and shown on Figures 1, 4, and S3. (DOC) [file pbio.1001455.s019.doc]

**Table S1: Cosinor statistical values related to rhythmic mRNA expression of genes coding for proteins involved in mRNA translation, TORC1 complex and ribosome biogenesis**

|  |  |  |  |  |  |  |  |  |  |
| --- | --- | --- | --- | --- | --- | --- | --- | --- | --- |
|  | Gene | p value | F[2,9] | Robustness (%) | Mesor | Amplitude | Acrophase (h) | Fold change |  |
|  | *Eif4e* | 0.01500 | 6.945 | 47.6 | 0.83 | 0.121 | 11.3 | 1.566 |  |
|  | *Eif4g1* | 0.00249 | 13.215 | 66.1 | 9.26 | 1.615 | 0.7 | 1.678 |  |
|  | *Eif4a2* | 0.00109 | 17.680 | 72.9 | 5.52 | 1.562 | 8.1 | 2.085 |  |
|  | *Eif4b* | 0.00043 | 24.953 | 79.6 | 8.85 | 2.263 | 5.7 | 1.835 |  |
|  | *Eif4ebp1* | 0.00051 | 23.339 | 78.4 | 1.88 | 0.860 | 9.1 | 3.126 |  |
|  | *Eif4ebp3* | 0.00022 | 32.418 | 83.7 | 5.82 | 4.998 | 15.8 | 7.647 |  |
|  |  |  |  |  |  |  |  |  |  |
|  | *mTor* | 0.00089 | 19.010 | 74.5 | 10.75 | 2.391 | 5.3 | 1.790 |  |
|  | *Raptor* | 0.00222 | 13.753 | 67.1 | 10.14 | 1.954 | 3.2 | 1.598 |  |
|  | *Map4k3* | 0.00022 | 32.061 | 83.6 | 1.09 | 0.258 | 6.1 | 1.628 |  |
|  | *Mnk2* | 0.00026 | 29.933 | 82.6 | 2.45 | 1.065 | 11.7 | 2.929 |  |
|  |  |  |  |  |  |  |  |  |  |
|  | *pre-45S* rRNA | 0.00025 | 30.395 | 82.8 | 13.90 | 3.630 | 9.0 | 1.913 |  |
|  | *Pre-Rpl23* | 0.00222 | 13.752 | 67.1 | 19.53 | 8.189 | 9.3 | 4.141 |  |
|  | *Pre-Rpl32* | 0.00011 | 43.290 | 87.4 | 15.17 | 5.865 | 8.8 | 2.480 |  |
|  | *Pre-Rpl34* | 0.00044 | 24.724 | 79.5 | 7.51 | 3.914 | 8.5 | 3.348 |  |
|  |  |  |  |  |  |  |  |  |  |
|  | *Ubf1* | 0.00025 | 30.734 | 83.0 | 1.81 | 0.577 | 6.1 | 2.211 |  |
|  |  |  |  |  |  |  |  |  |  |
