# Supplementary material for: The Circadian Clock Coordinates Ribosome Biogenesis
Source: PLoS Biol. 2013 Jan 3;11(1):e1001455. doi: 10.1371/journal.pbio.1001455 (PMC3536797; doi:10.1371/journal.pbio.1001455)
Supplement: Table S2 — Cosinor statistical values related to rhythmic expression and phosphorylations of proteins involved in mRNA translation, TORC1 complex, and ribosome biogenesis. A Cosinor statistical analysis was applied to the rhythmic datasets corresponding to the respective expression of the indicated proteins measured by Western blots quantification in WT mice and shown on Figures S1, S2, S11, and S12. (DOC) [file pbio.1001455.s020.doc]

**Table S2: Cosinor statistical values related to rhythmic phosphorylation and expression of protein involved in mRNA translation, cell signaling and ribosome biogenesis**

| Protein | p value | F[2,9] | Robustness (%) | Mesor | Amplitude | Acrophase (h) | Fold change |
| --- | --- | --- | --- | --- | --- | --- | --- |
| P-EIF4E | 0.01231 | 7.478 | 49.9 | 3.40 | 2.04 | 7.51 | 5.89 |
| P-EIF4G1 | 0.00185 | 14.662 | 68.7 | 4.21 | 3.68 | 16.73 | 9.69 |
| P-EIF4B | 0.01404 | 7.120 | 48.4 | 4.41 | 4.47 | 14.64 | 13.44 |
| P-4E-BP1 | 0.00488 | 10.439 | 59.8 | 25.06 | 22.43 | 13.68 | 64.81 |
| P-RPS6 | 0.00604 | 9.683 | 57.7 | 43.96 | 56.27 | 15.83 | 110.02 |
|  |  |  |  |  |  |  |  |
| P-TSC2 | 0.00212 | 13.996 | 67.6 | 4.76 | 3.43 | 7.85 | 10.57 |
| P-AKT | 0.00212 | 13.993 | 67.6 | 3.51 | 2.63 | 14.69 | 8.37 |
| P-ERK | 0.00151 | 15.746 | 70.4 | 5.60 | 4.45 | 7.33 | 11.04 |
| P-p90RSK | 0.00044 | 24.711 | 79.5 | 2.34 | 1.51 | 6.39 | 4.28 |
|  |  |  |  |  |  |  |  |
| Me-GTP 4E-BP1 | 0.00299 | 12.404 | 64.5 | 2.97 | 1.76 | 4.74 | 5.49 |
|  |  |  |  |  |  |  |  |
| RPL5 | 0.01036 | 7.970 | 51.9 | 2.13 | 0.66 | 12.52 | 3.00 |
| RPL23 | 0.04426 | 4.471 | 33.1 | 2.45 | 1.02 | 15.84 | 4.15 |
| RPL32 | 0.00007 | 52.586 | 89.5 | 4.01 | 2.38 | 14.51 | 6.78 |
| RPLP0 | 0.01075 | 7.864 | 51.5 | 3.57 | 2.20 | 10.93 | 7.57 |
|  |  |  |  |  |  |  |  |
| UBF1 | 0.00328 | 12.009 | 63.7 | 2.57 | 1.01 | 6.49 | 3.71 |
