# Supplementary material for: The Circadian Clock Coordinates Ribosome Biogenesis
Source: PLoS Biol. 2013 Jan 3;11(1):e1001455. doi: 10.1371/journal.pbio.1001455 (PMC3536797; doi:10.1371/journal.pbio.1001455)
Supplement: Table S4 — Functions of the genes presenting a rhythmic total/polysomal RNA ratio. Most of the genes found regulated at the translational level are known 5′-TOP containing genes. They include almost all the RP coding genes: 28 of the 32 small RP genes and 42 of the 47 large RP genes expressed in mouse [83] are found on the list. The list also includes known 5′-TOP mRNA encoding proteins involved in the regulation of translation: translation initiation factors of the class 2, 3, and 4, first class of translation elongation factors, and poly-A binding proteins [19]. In addition, the list contains genes encoding proteins involved at different steps of translational regulation and ribosome biogenesis: NPM1, a chaperone protein involved in ribosome assembly and rRNA maturation [84]; CCT4, a member of the chaperonin complex that plays a role in ribosome biogenesis [85]; TPT1, a guanine nucleotide exchanger that controls TORC1 activity through regulation of the RHEB GTPase [86]; IGBP1, a regulatory subunit of protein phosphatase 2A that modulates TORC1 activity [87]; PFDN5, a chaperone protein that modulates MYC activity [88]; a transcription factor involved in rRNA and RP mRNA transcription [89]; AHCY, a S-adenosyl homocysteine hydrolase that regulates translation also through modulation of MYC activity [90]; GNB2L1 or RACK1, a scaffold protein that interacts with and modulates ribosome activity [80]; UBA52, a protein constitutes by the fusion of a ribosomal protein and ubiquitin [91]; The remaining genes encode proteins with unknown function in translation regulation. (DOC) [file pbio.1001455.s022.doc]

**Table S4: Functions of the genes presenting a rhythmic total / polysomal RNA ratio**

| Ribosomes small subunits | Ribosomes large subunits | Translation initiation factors | Translation elongation factors | Role in ribosome biogenesis | Other functions |
| --- | --- | --- | --- | --- | --- |
| Rpsa | Rpl3 | Eif2a | Eef1a1 | Npm1 | Ercc6l |
| Rps2 | Rpl4 | Eif3e | Eef1a2 | Cct4 | Rabggtb |
| Rps3 | Rpl5 | Eif3f | Eef1b2 | Tpt1 | Sft2d2 |
| Rps3a | Rpl6 | Eif3h | Eef1d | Igbp1 | Cox7a2l |
| Rps4x | Rpl7 | Eif4a2 | Eef1g | Pfdn5 |  |
| Rps5 | Rpl7a | Eif4b | pabpc4 | Ahcy |  |
| Rps6 | Rpl8 |  |  | Gnb2l1 |  |
| Rps7 | Rpl9 |  |  | Uba52 |  |
| Rps8 | Rpl10 |  |  |  |  |
| Rps9 | Rpl10a |  |  |  |  |
| Rps10 | Rpl11 |  |  |  |  |
| Rps12 | Rpl12 |  |  |  |  |
| Rps14 | Rpl13 |  |  |  |  |
| Rps15 | Rpl13a |  |  |  |  |
| Rps15a | Rpl14 |  |  |  |  |
| Rps16 | Rpl15 |  |  |  |  |
| Rps17 | Rpl17 |  |  |  |  |
| Rps18 | Rpl18a |  |  |  |  |
| Rps19 | Rpl21 |  |  |  |  |
| Rps20 | Rpl22 |  |  |  |  |
| Rps21 | Rpl23 |  |  |  |  |
| Rps23 | Rpl23a |  |  |  |  |
| Rps24 | Rpl24 |  |  |  |  |
| Rps25 | Rpl26 |  |  |  |  |
| Rps26 | Rpl27 |  |  |  |  |
| Rps27 | Rpl27a |  |  |  |  |
| Rps27a | Rpl28 |  |  |  |  |
| Rps28 | Rpl29 |  |  |  |  |
|  | Rpl30 |  |  |  |  |
|  | Rpl31 |  |  |  |  |
|  | Rpl32 |  |  |  |  |
|  | Rpl34 |  |  |  |  |
|  | Rpl35 |  |  |  |  |
|  | Rpl35a |  |  |  |  |
|  | Rpl36 |  |  |  |  |
|  | Rpl36a |  |  |  |  |
|  | Rpl37 |  |  |  |  |
|  | Rpl37a |  |  |  |  |
|  | Rpl38 |  |  |  |  |
|  | Rpl39 |  |  |  |  |
|  | Rplp0 |  |  |  |  |
|  | Rplp1 |  |  |  |  |
