# Supplementary material for: The Circadian Clock Coordinates Ribosome Biogenesis
Source: PLoS Biol. 2013 Jan 3;11(1):e1001455. doi: 10.1371/journal.pbio.1001455 (PMC3536797; doi:10.1371/journal.pbio.1001455)
Supplement: Table S5 — Cosinor statistical values related to rhythmic mRNA expression of genes coding for proteins involved in mRNA translation, TORC1 complex, and ribosome biogenesis in WT and Cry1 / Cry2 KO mice. A Cosinor statistical analysis was applied to the rhythmic datasets corresponding to the respective expression of the indicated mRNA measured by quantitative PCR in WT and Cry1/Cry2 KO mice and shown on Figures 4, 5, and S14. (DOC) [file pbio.1001455.s023.doc]

**Table S5: Cosinor statistical values related to rhythmic mRNA expression of genes coding for proteins involved in mRNA translation, TORC1 complex and ribosome biogenesis in wild-type and *Cry1*/*Cry2*** KO mice

| Gene | Genotype | p value | F[2,9] | Robustness (%) | Mesor | Mesor p value | Amplitude | Acrophase (h) |
| --- | --- | --- | --- | --- | --- | --- | --- | --- |
| *Eif4e* | *WT* | 0.01500 | 6.945 | 47.6 | 8.28 | 0.00000 | 1.21 | 11.26 |
|  | *KO* | n.s. |  |  | 14.23 |  |  |  |
| *Eif4g1* | *WT* | 0.00249 | 13.215 | 66.1 | 9.26 | 0.00003 | 1.61 | 0.70 |
|  | *KO* | n.s. |  |  | 6.95 |  |  |  |
| *Eif4a2* | *WT* | 0.00109 | 17.680 | 72.9 | 5.52 | 0.00736 | 1.56 | 8.06 |
|  | *KO* | n.s. |  |  | 4.32 |  |  |  |
| *Eif4b* | *WT* | 0.00043 | 24.953 | 79.6 | 0.88 | 0.02120 | 0.23 | 5.75 |
|  | *KO* | n.s. |  |  | 0.74 |  |  |  |
| *Eif4ebp1* | *WT* | 0.00062 | 21.751 | 77.1 | 1.20 | 0.00000 | 0.55 | 9.20 |
|  | *KO* | n.s. |  |  | 3.51 |  |  |  |
| *Eif4ebp3* | *WT* | 0.00013 | 39.929 | 86.5 | 4.24 | n.s. | 3.75 | 15.82 |
|  | *KO* | 0.00583 | 9.806 | 58.1 | 4.84 |  | 1.87 | 9.36 |
| *mTor* | *WT* | 0.00089 | 19.010 | 74.5 | 10.75 | 0.00024 | 2.39 | 5.25 |
|  | *KO* | n.s. |  |  | 7.87 |  |  |  |
| *Raptor* | *WT* | 0.00222 | 13.753 | 67.1 | 10.14 | 0.00328 | 1.95 | 3.23 |
|  | *KO* | n.s. |  |  | 8.31 |  |  |  |
| *pre-45S* rRNA | *WT* | 0.00019 | 33.888 | 84.4 | 4.36 | 0.00022 | 1.29 | 9.12 |
|  | *KO* | 0.00525 | 10.172 | 59.1 | 2.91 |  | 0.60 | 9.42 |
| *Pre-Rpl23* | *WT* | 0.00571 | 9.877 | 58.3 | 2.46 | 0.00000 | 1.11 | 9.45 |
|  | *KO* | 0.00463 | 10.632 | 60.3 | 5.40 |  | 1.30 | 11.55 |
| *Pre-Rpl32* | *WT* | 0.00011 | 42.969 | 87.4 | 7.01 | 0.00003 | 2.72 | 8.77 |
|  | *KO* | 0.01468 | 7.002 | 47.8 | 12.83 |  | 3.34 | 12.97 |
| *Pre-Rpl34* | *WT* | 0.00068 | 20.978 | 76.5 | 8.41 | 0.00002 | 4.61 | 8.53 |
|  | *KO* | n.s. |  |  | 15.89 |  |  |  |
| *Ubf1* | *WT* | 0.00025 | 30.734 | 83.0 | 1.81 | 0.00002 | 0.58 | 6.14 |
|  | *KO* | n.s. |  |  | 1.08 |  |  |  |
