# Supplementary material for: The Circadian Clock Coordinates Ribosome Biogenesis
Source: PLoS Biol. 2013 Jan 3;11(1):e1001455. doi: 10.1371/journal.pbio.1001455 (PMC3536797; doi:10.1371/journal.pbio.1001455)
Supplement: Table S6 — Cosinor statistical values related to rhythmic mRNA expression of genes coding for proteins involved in mRNA translation, TORC1 complex and ribosome biogenesis in WT and Bmal1 KO mice. A Cosinor statistical analysis was applied to the rhythmic datasets corresponding to the respective expression of the indicated mRNA measured by quantitative PCR in WT and Bmal1 KO mice and shown on Figures 4, 5, and S15. (DOC) [file pbio.1001455.s024.doc]

**Table S6: Cosinor statistical values related to rhythmic mRNA expression of genes coding for proteins involved in mRNA translation, TORC1 complex and ribosome biogenesis in wild-type and *Bmal1* KO mice**

| Gene | Genotype | p value | F[2,9] | Robustness (%) | Mesor | Mesor p value | Amplitude | Acrophase (h) |
| --- | --- | --- | --- | --- | --- | --- | --- | --- |
| *Eif4e* | *WT* | 0.01748 | 6.551 | 45.7 | 2.21 | n.s. | 0.49 | 12.39 |
|  | *KO* | n.s. |  |  | 2.40 |  |  |  |
| *Eif4g1* | *WT* | 0.02316 | 5.868 | 42.1 | 10.36 | 0.00225 | 1.11 | 3.80 |
|  | *KO* | 0.04055 | 4.647 | 34.4 | 12.22 |  | 1.46 | 22.32 |
| *Eif4a2* | *WT* | 0.01083 | 7.842 | 51.4 | 13.82 | 0.00002 | 3.28 | 9.16 |
|  | *KO* | 0.00469 | 10.585 | 60.2 | 21.12 |  | 4.13 | 11.54 |
| *Eif4b* | *WT* | 0.03973 | 4.689 | 34.7 | 2.11 | 0.00022 | 0.48 | 8.79 |
|  | *KO* | 0.03240 | 5.116 | 37.6 | 3.08 |  | 0.57 | 12.29 |
| *Eif4ebp1* | *WT* | 0.04749 | 4.333 | 32.1 | 9.50 | 0.00768 | 3.28 | 11.84 |
|  | *KO* | 0.00734 | 9.030 | 55.7 | 5.47 |  | 3.63 | 12.96 |
| *Eif4ebp3* | *WT* | 0.00099 | 18.321 | 73.7 | 15.74 | n.s. | 14.77 | 16.98 |
|  | *KO* | 0.01965 | 6.260 | 44.2 | 19.17 |  | 12.18 | 15.47 |
| *mTor* | *WT* | 0.00317 | 12.153 | 64.0 | 4.04 | 0.01286 | 1.23 | 9.67 |
|  | *KO* | 0.00078 | 19.924 | 75.4 | 5.05 |  | 0.92 | 10.38 |
| *Raptor* | *WT* | 0.00450 | 10.745 | 60.6 | 3.56 | 0.00007 | 0.62 | 6.86 |
|  | *KO* | n.s. |  |  | 4.56 |  |  |  |
| *pre-45S* rRNA | *WT* | 0.00031 | 28.143 | 81.6 | 13.30 | 0.00000 | 4.40 | 9.84 |
|  | *KO* | 0.04012 | 4.669 | 34.6 | 20.43 |  | 1.98 | 1.73 |
| *Pre-Rpl23* | *WT* | 0.03388 | 5.020 | 37.0 | 6.43 | n.s. | 2.07 | 14.17 |
|  | *KO* | 0.00338 | 11.879 | 63.4 | 7.38 |  | 1.84 | 17.48 |
| *Pre-Rpl32* | *WT* | 0.03375 | 5.029 | 37.0 | 0.61 | n.s. | 0.27 | 13.85 |
|  | *KO* | 0.03433 | 4.993 | 36.8 | 0.76 |  | 0.14 | 17.97 |
| *Pre-Rpl34* | *WT* | 0.04662 | 4.369 | 32.3 | 0.79 | n.s. | 0.29 | 13.23 |
|  | *KO* | n.s. |  |  | 0.81 |  |  |  |
| *Ubf1* | *WT* | 0.00017 | 35.933 | 85.2 | 2.35 | 0.00002 | 0.62 | 7.49 |
|  | *KO* | n.s. |  |  | 3.23 |  |  |  |
