# Supplementary material for: The Circadian Clock Coordinates Ribosome Biogenesis
Source: PLoS Biol. 2013 Jan 3;11(1):e1001455. doi: 10.1371/journal.pbio.1001455 (PMC3536797; doi:10.1371/journal.pbio.1001455)
Supplement: Table S7 — Cosinor statistical values related to rhythmic expression and phosphorylation of proteins involved in mRNA translation, TORC1 complex and ribosome biogenesis in WT and Cry1 / Cry2 KO mice. A Cosinor statistical analysis was applied to the rhythmic datasets corresponding to the respective expression of the indicated proteins measured by Western blots quantification in WT and Cry1/Cry2 KO mice and shown on Figures S12 and S16. (DOC) [file pbio.1001455.s025.doc]

**Table S7: Cosinor statistical values related to rhythmic phosphorylation and expression of protein involved in mRNA translation, cell signaling and ribosome biogenesis in wild-type and *Cry1*/*Cry2* KO mice**

| Gene | Genotype | p value | F[2,9] | Robustness (%) | Mesor | mesor p value | Amplitude | Acrophase (h) |
| --- | --- | --- | --- | --- | --- | --- | --- | --- |
| P-EIF4E | *WT* | 0.00325 | 12.044 | 63.7 | 16.52 | n.s. | 13.57 | 8.30 |
|  | *KO* | n.s. |  |  | 19.62 |  |  |  |
| P-EIF4G1 | *WT* | 0.00365 | 11.559 | 62.6 | 54.61 | 0.09835 | 51.70 | 18.33 |
|  | *KO* | 0.00274 | 12.788 | 65.3 | 27.29 |  | 36.46 | 15.99 |
| P-RPS6 | *WT* | 0.00909 | 8.360 | 53.3 | 125.80 | n.s. | 157.26 | 15.88 |
|  | *KO* | 0.00603 | 9.686 | 57.7 | 181.56 |  | 176.00 | 12.99 |
| P-AKT | *WT* | 0.04074 | 4.638 | 34.3 | 23.67 | n.s. | 20.05 | 15.10 |
|  | *KO* | 0.01304 | 7.320 | 49.2 | 19.89 |  | 18.77 | 13.51 |
| P-ERK | *WT* | 0.03083 | 5.223 | 38.3 | 1.79 | 0.00008 | 0.56 | 4.63 |
|  | *KO* | n.s. |  |  | 3.27 |  |  |  |
| RPL5 | *WT* | 0.03941 | 5.215 | 38.2 | 3.02 | n.s. | 1.37 | 16.76 |
|  | *KO* | 0.00054 | 22.830 | 78.0 | 3.46 |  | 2.28 | 9.38 |
| RPL23 | *WT* | 0.04165 | 4.593 | 34.0 | 18.67 | 0.00123 | 13.70 | 17.15 |
|  | *KO* | 0.04366 | 4.499 | 33.3 | 36.04 |  | 7.96 | 0.09 |
| RPL32 | *WT* | 0.04843 | 4.294 | 31.8 | 5.71 | 0.00002 | 1.90 | 13.36 |
|  | *KO* | 0.02661 | 5.549 | 40.3 | 9.95 |  | 1.76 | 1.60 |
| RPLP0 | *WT* | 0.00112 | 17.533 | 72.8 | 2.68 | 0.00051 | 1.33 | 10.85 |
|  | *KO* | 0.01775 | 6.512 | 45.5 | 4.92 |  | 1.62 | 19.77 |
| UBF1 | *WT* | 0.00099 | 18.304 | 73.7 | 3.01 | 0.00005 | 0.97 | 8.63 |
|  | *KO* | n.s. |  |  | 1.68 |  |  |  |
