# Supplementary material for: The Circadian Clock Coordinates Ribosome Biogenesis
Source: PLoS Biol. 2013 Jan 3;11(1):e1001455. doi: 10.1371/journal.pbio.1001455 (PMC3536797; doi:10.1371/journal.pbio.1001455)
Supplement: Table S8 — Cosinor statistical values related to rhythmic expression and phosphorylation of proteins involved in mRNA translation, TORC1 complex, and ribosome biogenesis in WT and Bmal1 KO mice. A Cosinor statistical analysis was applied to the rhythmic datasets corresponding to the respective expression of the indicated proteins measured by Western blots quantification in WT and Bmal1 KO mice and shown on Figures S12 and S17. (DOC) [file pbio.1001455.s026.doc]

**Table S8: Cosinor statistical values related to rhythmic phosphorylation and expression of protein involved in mRNA translation, cell signaling and ribosome biogenesis in wild-type and *Bmal1* KO mice**

| Gene | Genotype | p value | F[2,9] | Robustness (%) | Mesor | mesor p value | Amplitude | Acrophase (h) |
| --- | --- | --- | --- | --- | --- | --- | --- | --- |
| P-EIF4E | *WT* | 0.03271 | 5.096 | 37.5 | 4.69 | n.s. | 1.28 | 6.45 |
|  | *KO* | 0.02043 | 6.166 | 43.7 | 5.16 |  | 1.74 | 12.38 |
| P-EIF4G1 | *WT* | 0.03883 | 4.736 | 35.0 | 2.33 | n.s. | 1.07 | 17.69 |
|  | *KO* | n.s. |  |  | 2.63 |  |  |  |
| P-RPS6 | *WT* | 0.00813 | 8.703 | 54.6 | 90.65 | 0.01235 | 94.45 | 17.79 |
|  | *KO* | n.s. |  |  | 38.66 |  |  |  |
| P-AKT | *WT* | 0.04379 | 4.493 | 33.3 | 17.83 | 0.00018 | 5.94 | 13.44 |
|  | *KO* | n.s. |  |  | 6.88 |  |  |  |
| P-ERK | *WT* | 0.00057 | 22.417 | 77.7 | 1.81 | n.s. | 0.75 | 8.97 |
|  | *KO* | n.s. |  |  | 1.74 |  |  |  |
| RPL5 | *WT* | 0.02301 | 5.883 | 42.2 | 2.05 | n.s. | 0.66 | 18.59 |
|  | *KO* | 0.00010 | 45.027 | 87.9 | 3.06 |  | 1.47 | 5.66 |
| RPL23 | *WT* | 0.04263 | 4.547 | 33.7 | 2.02 | n.s. | 0.49 | 18.84 |
|  | *KO* | 0.00474 | 10.550 | 60.1 | 2.49 |  | 1.19 | 7.00 |
| RPL32 | *WT* | 0.04760 | 4.328 | 32.0 | 2.32 | 0.00036 | 0.72 | 19.11 |
|  | *KO* | 0.00056 | 22.488 | 77.8 | 3.93 |  | 1.34 | 7.83 |
| RPLP0 | *WT* | 0.04692 | 4.356 | 32.3 | 2.76 | n.s. | 0.82 | 21.80 |
|  | *KO* | 0.00520 | 10.211 | 59.2 | 2.77 |  | 0.99 | 6.63 |
| UBF1 | *WT* | 0.02444 | 5.743 | 41.4 | 6.07 | 0.00013 | 3.33 | 7.03 |
|  | *KO* | n.s. |  |  | 10.92 |  |  |  |
