# Supplementary material for: The Circadian Clock Coordinates Ribosome Biogenesis
Source: PLoS Biol. 2013 Jan 3;11(1):e1001455. doi: 10.1371/journal.pbio.1001455 (PMC3536797; doi:10.1371/journal.pbio.1001455)
Supplement: Table S9 — Taqman probes used for real-time PCR (Applied Biosystems). (DOC) [file pbio.1001455.s027.doc]

**Table S9: Taqman probes used for real-time PCR (Applied Biosystems)**

| **Gene** | **Probe reference** |
| --- | --- |
| *Gapdh* | Mm 99999915_g1 |
| *28S rRNA* | Mm 03682676_s1 |
| *Eif4e* | Mm 00725633_s1 |
| *Eif4g1* | Mm 00524099_m1 |
| *Eif4a2* | Mm 00834357_g1 |
| *Eif4b* | Mm 00778003_s1 |
| *Eif4ebp1* | Mm 01620026_g1 |
| *Eif4ebp3* | Mm 01406408_m1 |
| *Rpl23* | Mm 00787512_s1 |
| *Rpl32* | Mm 02528467_g1 |
| *Rpl34* | Mm 01318199_g1 |
| *mTor* | Mm 00444968_m1 |
| *Raptor* | Mm 00712676_m1 |
| *Map4k3* | Mm 01232993_m1 |
| *Mknk2* | Mm 00458026_m1 |
| *Ubf1* | Mm 00456972_m1 |
