# Supplementary material for: The Circadian Clock Coordinates Ribosome Biogenesis
Source: PLoS Biol. 2013 Jan 3;11(1):e1001455. doi: 10.1371/journal.pbio.1001455 (PMC3536797; doi:10.1371/journal.pbio.1001455)
Supplement: Table S10 — Sequences of the primers used for SYBR Green real-time PCR. (DOC) [file pbio.1001455.s028.doc]

**Table S10: Sequences of the primers used for SYBR® Green real-time PCR**

| **Gene** | **Forward primer** | **Reverse primer** |
| --- | --- | --- |
| *Gapdh* | CATGGCCTTCCGTGTTCCTA | CCTGCTCTTCCGTGTTCCTA |
| *45S rRNA* | GCTGCCTCACCAGTCTTTCT | GCAAGACCCAAACACACACA |
| *Rpl23 intron 3* | ATTGATGAACACGGCAAACA | GAGTTCGAGACCGAGACCAG |
| *Rpl32 intron 3* | TACAGCAGCAGTCCATGAGG | CACCCCAGGACTCTTTACCA |
| *Rpl34 intron 3* | CCTGCCCTGTTTGTGGTAGT | TGGAAATCTTTTCCGTTTGC |
