# Supplementary material for: The Circadian Clock Coordinates Ribosome Biogenesis
Source: PLoS Biol. 2013 Jan 3;11(1):e1001455. doi: 10.1371/journal.pbio.1001455 (PMC3536797; doi:10.1371/journal.pbio.1001455)
Supplement: Table S11 — References of the antibodies used for Western blotting [92],[93] . (DOC) [file pbio.1001455.s029.doc]

**Table S11: References of the antibodies used for Western blotting**
